# Transcriptional analysis of abdominal fat in chickens divergently selected on bodyweight at two ages reveals novel mechanisms controlling adiposity: validating visceral adipose tissue as a dynamic endocrine and metabolic organ

**DOI:** 10.1186/s12864-017-4035-5

**Published:** 2017-08-16

**Authors:** C. W. Resnyk, W. Carré, X. Wang, T. E. Porter, J. Simon, E. Le Bihan-Duval, M. J. Duclos, S. E. Aggrey, L. A. Cogburn

**Affiliations:** 1 Department of Animal and Food Sciences, University of Delaware, Newark, DE 19716 USA; 2 Laboratoire de Génétique Moléculaire et Génomique, CHU Pontchaillou, 35033 Rennes, France; 3 Department of Biological Sciences, Tennessee State University, Nashville, TN 37209 USA; 4 Department of Animal and Avian Sciences, University of Maryland, College Park, MD 20742 USA; 5 UR83 Recherches Avicoles, Institut National de la Recherche Agronomique (INRA), F-37380 Nouzilly, France; 6 Department of Poultry Science, University of Georgia, Athens, GA 30602 USA

**Keywords:** Divergent genetic selection, Gene expression, Microarray analysis, RNA-Seq analysis, Adiposity, Lipogenesis, Transcriptional regulation, Hemostasis genes, Endocrine signaling

## Abstract

**Background:**

Decades of intensive genetic selection in the domestic chicken (*Gallus gallus domesticus*) have enabled the remarkable rapid growth of today’s broiler (meat-type) chickens. However, this enhanced growth rate was accompanied by several unfavorable traits (i.e., increased visceral fatness, leg weakness, and disorders of metabolism and reproduction). The present descriptive analysis of the abdominal fat transcriptome aimed to identify functional genes and biological pathways that likely contribute to an extreme difference in visceral fatness of divergently selected broiler chickens.

**Methods:**

We used the Del-Mar 14 K Chicken Integrated Systems microarray to take time-course snapshots of global gene transcription in abdominal fat of juvenile [1-11 weeks of age (wk)] chickens divergently selected on bodyweight at two ages (8 and 36 wk). Further, a RNA sequencing analysis was completed on the same abdominal fat samples taken from high-growth (HG) and low-growth (LG) cockerels at 7 wk, the age with the greatest divergence in body weight (3.2-fold) and visceral fatness (19.6-fold).

**Results:**

Time-course microarray analysis revealed 312 differentially expressed genes (FDR ≤ 0.05) as the main effect of genotype (HG versus LG), 718 genes in the interaction of age and genotype, and 2918 genes as the main effect of age. The RNA sequencing analysis identified 2410 differentially expressed genes in abdominal fat of HG versus LG chickens at 7 wk. The HG chickens are fatter and over-express numerous genes that support higher rates of visceral adipogenesis and lipogenesis. In abdominal fat of LG chickens, we found higher expression of many genes involved in hemostasis, energy catabolism and endocrine signaling, which likely contribute to their leaner phenotype and slower growth. Many transcription factors and their direct target genes identified in HG and LG chickens could be involved in their divergence in adiposity and growth rate.

**Conclusions:**

The present analyses of the visceral fat transcriptome in chickens divergently selected for a large difference in growth rate and abdominal fatness clearly demonstrate that abdominal fat is a very dynamic metabolic and endocrine organ in the chicken. The HG chickens overexpress many transcription factors and their direct target genes, which should enhance in situ lipogenesis and ultimately adiposity. Our observation of enhanced expression of hemostasis and endocrine-signaling genes in diminished abdominal fat of LG cockerels provides insight into genetic mechanisms involved in divergence of abdominal fatness and somatic growth in avian and perhaps mammalian species, including humans.

**Electronic supplementary material:**

The online version of this article (doi:10.1186/s12864-017-4035-5) contains supplementary material, which is available to authorized users.

## Background

The domestic chicken (*Gallus gallus domesticus*) is a widely used biomedical model and serves as a major source of high-quality dietary protein for humans. Decades of intensive genetic selection have led to the remarkable growth rate and feed efficiency of commercial broiler chickens. However, this rigorous genetic selection for growth rate has led to increased adiposity, skeletal abnormalities, and disorders of metabolism and reproduction [1–4]. Few studies have attempted to identify biological pathways and gene networks that promote abdominal fatness and related metabolic disorders in the chicken. Previous studies of global gene transcription in different models of chicken growth have concentrated on either skeletal muscle [5] or the hypothalamus [6] for identification of candidate genes responsible for differences in growth rate. Another study compared intra-muscular adipose tissue between two lines of chickens selected for fast growth or slow growth [7]. This microarray analysis showed that expression of several differentially expressed (DE) genes was correlated with increased or decreased growth of breast muscle and intramuscular fat, which suggests that adipose tissue per se could regulate the rate of muscle growth, albeit no mechanisms related to abdominal fatness were uncovered.

A large volume of research has been published on growth and metabolic characteristics of the Virginia Tech (VT) population [8, 9] of chickens, which were divergently selected on body weight at 56 days of age (8 wk) [10–19]. The VT low-weight strain (LWS) chickens exhibit diminished growth, anorexia [15], impaired endocrine signaling [13, 15, 19, 20], and higher expression of genes involved in catabolism of lipid [17]. A genomic scan of the VT high-weight (HWS) and low-weight (LWS) selected chickens revealed several quantitative trait loci (QTL) and their associated polymorphic genes related to divergent selection for body weight at 8 wk [9]. Among the candidate genes identified by combined analyses of genome sequence variation in the VT HWS and LWS chickens were glucagon (*GCG*), insulin-like growth factor binding protein-2 (*IGFBP2*) and endothelin-1 (*EDN1*). Furthermore, we found that these three genes (*GCG, IGFBP2* and *END1*) were differentially expressed in abdominal fat of genetically fat (FL) and lean (LL) chickens [21, 22]. Our transcriptional study of abdominal fat in the FL and LL chickens also revealed over-expression of numerous lipogenic genes in the FL, which suggests that adipose tissue has a more significant involvement in the synthesis and metabolism of lipids than previously thought [21]. More importantly, we discovered over-expression of an unusually large number of genes involved in hemostasis, endocrine signaling and lipid catabolism [22] in the diminished abdominal fat of LL chickens.

The present study focuses on the transcriptional analysis of abdominal fat in a random-bred population of *Bresse-Pile* (meat-type) chickens, which were divergently selected by Ricard [23] for either high growth (HG) or low growth (LG) body weight at two developmental ages [juvenile (8 wk) and adult (36 wk)]. The diverse growth curves [24] and muscle growth patterns [25–29] of the HG and LG chickens have been described in detail. These unique growth models were chosen for our original functional genomics project aimed at identification of genomic regions and gene networks, which control growth and metabolism of the broiler chicken [30–32]. Despite genetic selection for a large difference in bodyweight (3.2-fold at 7 wk), these divergent lines exhibit an even greater difference in abdominal fat weight (19.6-fold at 7 wk). Furthermore, we have published several papers from quantitative trait loci (QTL) analyses using the F2 resource population created from an intercross of HG and LG chickens [33–36]. These genetic analyses have revealed numerous genomic loci containing positional candidate genes associated with several metabolic and growth traits. Another study of this unique F2 resource population has identified a *cis* expression QTL (eQTL) controlling β-carotene 15, 15′-monooxygenase (*BCMO1*) expression, which consequently determines the extent of yellow coloring in chicken breast muscle, an important meat quality trait [37]. The LG chickens, which carry the inactive *BCMO1* gene, exhibit a slower growth rate, greatly-reduced abdominal (visceral) fatness, lower plasma insulin, and hyperglycemia when compared to the HG chickens [34].

Thus, the present study aimed to identify differential gene expression in abdominal fat of HG and LG chickens, which show a greater divergence in abdominal fatness that is incidental to their selection on body weight at two ages (juvenile and adult). Our analyses reveal overexpression of genes in abdominal fat of the HG birds that are involved in increased adiposity, including transcriptional regulators and metabolic (lipogenic) enzymes, throughout juvenile development (1-11 wk). Conversely, LG chickens shown up-regulation of several energy generating processes (i.e., peroxisomal β-oxidation, mitochondrial β-oxidation, ketogenesis and oxidative phosphorylation) early in juvenile development which are likely responsible for their extreme leanness. The RNA-Seq analysis of abdominal fat at 7 wk. revealed up-regulation of several hemostatic factors in the LG cockerels that could contribute to their extreme leanness. Furthermore, this transcriptional study of visceral adiposity in HG and LG meat-type (*Bresse-Pile*) chickens also serves as cross-validation of abdominal fat as a dynamic endocrine and metabolic organ as indicated in our genetically fat (FL) and lean (LL) chicken lines [21, 22].

## Methods

### Animal management and tissue preparation

The chickens used in this study were divergently selected from a population of *Bresse-Pile* (meat-type) chickens by Ricard [23, 38] for an extreme difference in body weight at two ages: 8 (juvenile) and 36 (adult) weeks of age (wk). Chickens were bred and raised at INRA UE1295 Pôle d’Expérimentation Avicole de Tours, F-37380, Nouzilly, France. At hatching, HG and LG cockerels were wing-banded and vaccinated against Marek’s disease virus. Birds were provided with ad libitum access to water and fed a conventional starter ration (22% crude protein and 3050 kcal ME/kg) from hatching to 3 wk and then with a grower pelleted ration from 3 to 11 wk. (20% crude protein, and 3100 kcal). The HG birds were separated from LG birds for the first 3 weeks (at which time LG chickens were provided crushed feed pellets) to increase early survival of the LG; afterwards, both lines were placed together and raised in floor pens (4.4 m × 3.9 m). Continuous incandescent light was provided for the first two days followed by a maintenance of a 14 h light /10 h dark cycle (14 L:10D). Infrared gas heaters provided supplemental heat and the ambient temperature was decreased progressively from 32 °C at hatching, until 22 °C was reached at 22 days. At 1, 3 5, 7, 9 and 11 wk, eight fed cockerels from each genetic line (HG and LG) were randomly selected, weighed and bled into heparinized syringes prior to cervical dislocation, and the excision and weighing of abdominal fat mass. Abdominal adipose tissue samples were immediately snap frozen in liquid nitrogen and stored at −75° C until further processing for RNA analysis.

## Transcriptional analysis

### RNA extraction

Abdominal fat aliquots from forty-eight individuals (4 HG and 4 LG per age at 1, 3, 5, 7, 9 and 11 wk) were homogenized and total cellular RNA extracted using guanidine thiocyanate and CsCl gradient purification [39], followed by DNase I treatment. The quality of RNA was determined with an RNA 6000 Nano Assay kit and the Model 2100 Bioanalyzer (Agilent Technologies; Palo Alto, CA). All samples used for RNA analyses had an RNA integrity number (RIN) greater than 9.0.

### Microarray analysis and statistical analysis of microarray data

The Del-Mar 14 K Chicken Integrated Systems Microarrays (Geo Platform # GPL1731) described earlier [31] were used for transcriptional profiling of four abdominal fat samples from each genotype (HG and LG), across 11 weeks of juvenile development (48 total individuals). Methods used for microarray preparation including labeling, hybridization, and image acquisition were described earlier [21]. Briefly, twenty-four Del-Mar 14 K Chicken Integrated Systems Microarrays were hybridized with 48 labeled samples using a balanced block design [40], where half of the birds from each genotype and age were labeled with Alexa Flour® 647 (red dye) and the other half with Alexa Flour® 555 (green dye); see Additional file 1 for experimental design. These hybridized microarrays were scanned with a GenePix 4000B scanner using GenePix Pro 4.1 software (Molecular Devices, Union City, CA) at wavelengths of 635 nm (Alexa® 647-labeling) and 532 nm (Alexa® 555-labeling) producing a combined TIFF image file for each slide. Laser power was set at 100% with the photomultiplier tube (PMT) setting adjusted for each scan producing a PMT count near unity. All slides were checked manually for quality, and all spots with inadequacies in signal, background or morphology were eliminated. The image analysis results were merged with Excel files (in GPR format) containing clone identification, spot location on slide, and most current gene name/function (based on BLAST score). The gene list were first annotated by clone ID, GenBank ID as determined by BLASTN or BLASTX analysis using the GeneBase function on our laboratory website [41].

The GPR files were used to determine differential expression in abdominal fat of HG and LG chickens. Log2 transformed median intensity values (for each dye) were normalized using a global LOWESS transformation (without background subtraction) to remove dye bias within microarray [42]. A two-way ANOVA was used to determine main effects of age (A) and genotype (G), and interactions between age and genotype (A x G); differences between genotypes at each age were also determined. The Benjamini-Hochberg procedure [43] was used to control experiment-wise false discovery rate (FDR) associated with multiple testing. The differently-expressed (DE) gene lists from the time course (1-11 wk) microarray analysis were functionally annotated using the GeneBase function on our laboratory website [41] and finally the Ingenuity Knowledge Base [44]. Expression values of 25 DE genes at 7 wk were retrieved from the microarray analysis for comparison across the three methods. For these 25 genes, a Student’s T-test was used to determine significant differences between genotypes.

### RNA-sequencing and statistical analyses

The same eight RNA samples (4 HG and 4 LG at 7 wk), originally used for the microarray analysis, were also used for construction of indexed (bar-coded) sequencing libraries. Libraries were made from 2 μg of total adipose RNA with the Illumina TruSeq® Stranded mRNA library preparation kit following standard Illumina protocols. All eight barcoded libraries were pooled and paired-end sequenced (101-bp reads) in duplicate lanes on an Illumina HiSeq 2000 Sequencing System (Illumina, Inc., San Diego, CA) at the Delaware Biotechnology Institute, University of Delaware (Newark, DE).

Sequences were trimmed for quality using a combination of custom Perl scripts and Btrim64 software [45]. Boxplot graphing of pre-and-post trimming reads confirmed the absence of outlier samples based on read count. After trimming, reads were mapped to the chicken genome assembly Galgal4.0 (down-loaded from Ensembl) using Tophat (version 1.3.3), followed by assembly and quantitation using Cufflinks software (v1.3.0). The fragments per kilobase of exon per million fragments mapped (FPKM) threshold for detection of a gene was set at FPKM > 0.5. The resulting gtf files were merged with cuffmerge, and differential expression was assessed using Cuffdiff. The two-sided *P*-value was corrected using the false discovery rate (FDR) which accounts for multiple testing procedures [43]. Genes with a FDR adjusted *P*-value (*P ≤* 0.05) and fold change ≥ 1.2 were considered to be differentially expressed (DE) transcripts. This detection threshold was based on our extensive transcriptional analyses of multiple tissues in the chicken.

### Ingenuity Pathway Analysis of differentially expressed genes from microarray and RNA-Seq analyses

 Differentially-expressed (DE) gene datasets from the time-course microarray analyses were submitted to Ingenuity® Pathway Analysis (IPA) [44] for functional annotation as “Analysis Ready” (AR) genes according to annotated mammalian genes and proteins accrued in the Ingenuity® Knowledge Base. The unique and commonly shared (intersect) gene sets were then used for Ingenuity Up-Stream Regulator analysis to identify transcription factors and their direct target genes.

### Quantitative RT-PCR analysis

Candidate DE genes were selected for verification of expression by quantitative RT-PCR (qRT-PCR) analysis from both the time-course microarray analysis (1-11 wk) and RNA-Seq analysis (7 wk). Superscript III reverse transcriptase (Invitrogen) and an oligo (dT) primer were used to prepare cDNA from 1 μg of RNA. Primers were designed using Primer Express v2.0 software (Applied Biosystems, Foster City, CA). Detailed information for each primer pair including gene name, gene symbol, primer sequences (forward and reverse), GenBank accession number and amplicon size is provided in Additional file 2.

The qRT-PCR assays were performed in an ABI Prism Sequence Detection System 7900HT using 50 ng of cDNA, Power SYBR green PCR master mix (Applied Biosystems, Foster City, CA) and 400 nM of each primer (forward and reverse; Sigma-Aldrich, St. Louis, MO) in duplicate wells. Disassociation curves were analyzed to confirm specific amplification and to verify absence of primer dimers. Resulting PCR products were subjected to agarose gel electrophoresis to compare PCR product size to expected amplicon size. To verify gene expression from the microarray and RNA-Seq analyses, the cycle time (Ct) for each sample was normalized to the corresponding sample geometric mean of two housekeeping genes: cytochrome c oxidase subunit VIIa polypeptide 2 like (*COX7A2L*) and ribosomal protein L14 (*RPL14*). The housekeeping genes were selected based on invariability in the microarray and RNA-Seq analyses. Their stable expression in qRT-PCR analysis was determined by Biogazelle qbase+ software [46]. The 2^-(∆∆Ct)^ formula was used to calculate relative transcript abundance [47]. The statistical analysis was performed using a general linear model procedure in SAS v9.3 and differences between genotypes at each age were determined using Tukey’s multiple comparisons test. For genes only analyzed at 7 wk., a Student’s T-test was used to detrmine differential expression. The significance level for statistical analysis was set at *P* ≤ 0.05.

### Independent bioinformatic analysis of RNA-Seq analyses of abdominal fat across four divergent genotypes

An independent analysis was conducted on two deposited RNA-Seq datasets of abdominal fat (7 wk) in four distinct genotypes/phenotypes, which were created by divergent genetic selection on juvenile and adult BW (HG vs. LG; NCBI GEO Series Accession # GSE49121) or abdominal fatness at the same BW [fat line (FL) vs. lean line (LL); NCBI GEO Series Accession # GSE42980)]. The systems biology analysis of these two RNA-Seq datasets was performed by the Animal Systems Biology Analysis and Modeling Center (ASBAMC), University of California at Davis, CA. The components of the custom bioinformatics pipeline used for systems biology analysis of domestic animals are described in detail on the project website [48]. The main purpose of this independent meta-analysis of abdominal fat transcriptomes across four distinct genotypes (HG-LG; FL-LL) at the same age (7 wk) was to identify a set of common and unique DE gene across the four genotypes for further functional and pathway analyses using IPA.

## Results

### Phenotypic measurements

**Fig. 1:**
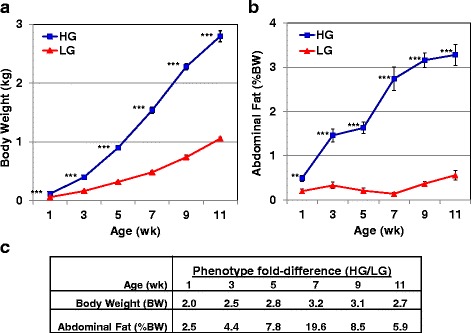
Phenotypic measurements of juvenile HG and LG chickens. Average body weight (**a**) of HG (blue squares) and LG (red triangles) cockerels at six ages (1-11 wk). Each symbol represents the mean ± SE of 8 individual chickens from each genotype. Average abdominal fat content (**b**) (% bodyweight, %BW) of 8 individual birds from the HG and LG chickens; four chickens per age and genotype were randomly selected for transcriptional analyses. Significant differences between genotypes at each age were determined using a one-way analysis of variance (ANOVA) and Tukey’s multiple comparisons procedure at a significance level of *P*≤0.01 (**) or *P*≤0.001 (***). Fold differences between genotypes (HG/LG) in body weight (kg) and abdominal fat (%BW) from 1 to 11 wk. (**c**). The maximum divergence in body weight and abdominal fatness occurs at 7 wk., which was the age selected for deeper RNA-Seq analysis

Body weight (BW) and relative abdominal fatness (%BW) of juvenile HG and LG chickens are presented in Fig. 1. On average, the HG cockerels were 2.7-fold heavier (*P*≤0.001; Fig. 1-a) and 8-fold fatter (*P*≤0.001; Fig. 1-b) than LG during juvenile development (1-11 wk). The greatest difference in BW and abdominal fat weight (%BW) was observed at 7 wk. where there were large differences (3.2- and 19.6-fold, respectively) between genetic lines (Fig. 1-c).

### Microarray and RNA-Seq analyses of abdominal fat gene expression

Differentially-expressed (DE) genes were defined as those having a significant adjusted *P*-value and false discovery rate (FDR≤0.05). Statistical analysis of the time-course microarray study provided significant gene sets from three contrasts: the main effect of genotype (312) DE genes) or age (2918 DE genes), and the interaction between genotype and age (718 DE genes). These annotated DE gene sets from the time-course microarray study are presented as HG/LG expression ratios in Additional file 3. These DE gene sets were used as input files for IPA and functionally annotated as “Analysis Ready” (AR) DE genes according to the Ingenuity Knowledge Base, which is largely based on annotations accrued from the human and murine biomedical literature.

RNA sequencing of abdominal fat at 7 wk. yielded 65.8 million (M) paired-end (101 bp) reads in HG cockerels and 66.6 M paired-end reads in the LG (Table 1). The percentage of reads mapped to chicken transcripts was 80.7% for the HG and 82.6% for the LG birds. A power analysis (Additional file 4) was conducted on the RNA-Seq dataset, using the web-based software program “Scotty” [49, 50], to demonstrate sufficient biological sample size and sequencing depth for detection of DE genes. Power was calculated using the average of 50 M reads per sample at three levels of fold-change (≥1.5-, 2-, or 3-fold change) between HG and LG chickens at a significance of *P*≤0.05. The “Scotty” program also provided a hierarchical cluster analysis using the Spearman correlation as the distance metric to demonstrate relatedness among the eight individual (4 HG and 4 LG) birds used for RNA-Seq analysis of abdominal fat at 7 wk. The 4 HG and 4 LG abdominal fat samples are tightly clustered according to their genotype. Statistical analysis of the RNA-Seq dataset identified 2410 DE (FDR ≤ 0.05) genes in abdominal fat of HG versus LG (HG/LG log2 expression ratio) chickens at 7 wk. (Additional file 5).

**Table 1:** Summary of reads mapped from RNA-Seq analysis of HG and LG abdominal fat (7 wk)

	Bird ID	Paired-End Reads	Reads Mapped (%)	Reads Unmapped (%)	Genes (FPKM > 0.5)
HG	1536	64,372,528	58,450,255 (91%)	5,922,272 (9%)	14,171
	1572	72,993,942	54,526,474 (75%)	18,467,467 (25%)	14,086
	1759	72,258,015	54,988,349 (76%)	17,269,665 (24%)	14,121
	1807	53,773,174	44,524,188 (83%)	9,248,985 (17%)	14,251
LG	1890	51,157,100	42,460,393 (83%)	8,696,707 (17%)	14,241
	1923	71,021,405	58,450,616 (82%)	12,570,788 (18%)	14,449
	5629	75,046,371	62,063,348 (83%)	12,983,022 (17%)	14,465
	5678	69,207,601	57,027063 (82%)	12,180,537 (18%)	14,362

Two replicate lanes of 8 multiplexed HG and LG abdominal fat samples were paired-end (101 bp) sequenced in an Illumina HiSeq 2000 sequencer. The percentage of mapped and unmapped reads is shown in parenthesis. The threshold for gene detection was set at greater than 0.5 fragments per kilobase of exon per million fragments mapped (FPKM > 0.5). Differential (DE) expression of a gene was determined by statistical difference after adjustment for a false discovery rate of FDR ≤ 0.05. The DE genes used for Ingenuity Pathways Analysis were considered analysis ready (AR) if annotated according to the Ingenuity Knowledge Base, accrued from human and murine models in the biomedical literature

**Fig. 2:**
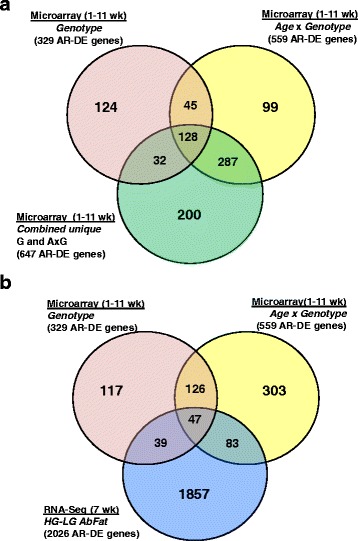
Venn diagram of “Analysis Ready” (AR) and differentially-expressed (DE) gene sets from the time-course (1-11 wk) microarray analysis (**a**) and RNA-seq analysis (**b**) of abdominal fat in HG and LG cockerels (7 wk). Three statistical contrasts were made for the microarray analysis: the main effect (FDR ≤ 0.05) of genotype (HG vs. LG) across 6 ages (1-11 wk), age x genotype interaction and the main effect of age. An additional non-redundant gene set (combined-unique, 647 AR-DE genes), used for Ingenuity® Pathway Analysis (IPA), was assembled by combining the genotype (329 AR-DE genes) and age x genotype (559 AR-DE genes) datasets, then removing and duplicate genes (cDNAs) printed on the 14 K Del-Mar chicken microarray. The Venn diagrams represent the number of AR-DE genes annotated in the Ingenuity® Knowledge Base. The common genes shown in intersects were also combined and used for IPA. The RNA-seq analysis provided 2026 AR-DE genes (FDR ≤ 0.05), which were used for IPA and comparison with time-course microarray datasets (**b**)

From the time-course microarray analysis, IPA annotated 329 “Analysis Ready” (AR)-DE genes from the main effect of genotype, 559 AR-DE genes from the interaction of genotype x age, and 647 AR-DE genes in a non-redundant dataset combined from the main effect of genotype and genotype x age interaction (Fig. 2-a). The second Venn diagram (Fig. 2-b) presents unique and commonly shared AR-DE genes from the time-course microarray study (genotype and age x genotype interaction) and 2026 AR-DE genes from RNA-Seq analysis of abdominal fat in HG and LG chickens at 7 wk. The number of DE genes considered by IPA as “Analysis Ready” (AR-DE) is shown in parenthesis.The Venn diagram (Fig. 2-a) presents unique and commonly shared DE gene sets found in HG and LG abdominal fat from the time-course microarray study. Only “Analysis Ready” (AR) gene sets, annotated with the Ingenuity Knowledge Base, are represented in the Venn diagram. An additional non-redundant gene set (combined-unique, 647 DE genes) was assembled by combining the genotype (329 DE genes) and age x genotype (559 DE genes) data sets and removing duplicated genes (cDNAs) printed on the microarray. A fourth dataset of 492 commonly-shared DE genes, found in these intersects, was also used for Ingenuity® Pathway Analysis (IPA). This commonly-shared DE gene dataset seems highly enriched with genes controlling the divergence in growth and abdominal fatness traits in HG and LG cockerels.

The RNA-Seq analysis (Fig. 2-b) of abdominal fat from the same 4 HG and 4 LG birds at 7 wk. provided 2026 DE genes that were annotated by IPA as “Analysis Ready” (AR). The intersection of the genotype and age x genotype datasets from the microarray study shows 126 commonly shared AR-DE genes. The RNA-Seq AR-DE gene set shared 86 common genes with the main effect of genotype AR-DE genes, and another 130 genes in common with the age x genotype AR-DE gene set. Only 47 AR-DE genes were shared among all three DE gene sets, derived from microarray and RNA-Seq analyses.

### IPA of gene interaction networks and functional pathways

#### Time-course microarray data analysis of abdominal fat in HG and LG cockerels (1-11 wk)

Significant gene (FDR≤0.05) lists from the microarray analysis were first annotated using the GeneBase tool on our laboratory website [41], which provides protein IDs (from GenBank or Swiss-Prot) derived from BLASTX/BLASTN analysis of 18,240 cDNA probes printed on the Del-Mar 14 K chicken array. Annotated microarray data files (Fig. 2-a), containing the *Ensembl* protein ID and log2 fold-difference for each gene, were submitted for Ingenuity® Pathway Analysis [44]. IPA provides functional annotation from the Ingenuity Knowledge Base [indicated as “Analysis Ready” (AR)], mapping to canonical metabolic/regulatory pathways, gene interaction networks and Ingenuity® Upstream Regulator Analysis, which provides transcription factor interaction networks and predicted interactions between transcription factors and their target genes.

A summary of the IPA of the time-course (1-11 wk) microarray dataset of 647 unique AR-DE genes condensed from the main effect of genotype and interaction of genotype x age datasets is presented in Table 2. The top five canonical pathways identified by microarray analysis were “Oxidative Phosphorylation” (19 AR-DE genes; Additional file 6), “Mitochondrial Dysfunction” (22 AR-DE genes), “Eukaryotic translation initiation factor (EIF2) Signaling” (21 AR-DE genes), “NRF2-mediated Oxidative Stress Response” (19 AR-DE genes), and “Phagosome Maturation” (13 AR-DE genes). The top five “Upstream Regulators” were TP53 (105 direct target genes), PPARA (50 direct targets), MYC (78 direct targets), PPARG (45 direct targets), and MYCN (32 direct targets). Ingenuity Upstream Regulator Analysis identified 22 DE transcription factors from the time-course microarray study; 8 transcription factors were up-regulated in the HG cockerels, whereas 14 upstream regulators were more abundant in LG abdominal fat. The top five “Molecular and Cellular Functions” identified by IPA from the microarray DE gene set included “Cellular Growth and Proliferation” (285 AR-DE genes, “Cellular Movement” (171 AR-DE genes), “Lipid Metabolism” (129 AR-DE genes), “Molecular Transport” (176 AR-DE genes), and “Small Molecule Biochemistry” (163 AR-DE genes). The top five IPA categories related to “Physiological System Development and Function” were “Cardiovascular System” (107 AR-DE genes), “Organismal Development” (181 AR-DE genes), “Organismal Survival” (175 AR-DE genes), “Hematological System” (123 AR-DE genes), and “Immune Cell Trafficking” (72 AR-DE genes). The top 10 up-regulated genes and down-regulated genes in abdominal fat of juvenile HG and LG chickens (1-11 wk) are also presented in Table 2.

**Table 2:** IPA summary of microarray analysis of abdominal fat in HG and LG cockerels (1-11 wk)

Top Canonical Pathways	*p*-value	Overlap	Ratio
Oxidative Phosphorylation	7.88E-10	17.4%	19/109
Mitochondrial Dysfunction	1.37E-08	12.9%	22/171
EIF2 Signaling	2.33E-07	11.4%	21/185
NRF2-mediated Oxidative Stress Response	2.84E-06	10.6%	19/180
Phagosome maturation	7.93E-05	10.8%	13/120
Top Upstream Regulators	*p*-value of overlap		# Target genes
TP53	2.92E-21		105
PPARA	1.46E-18		50
MYC	2.75E-16		78
PPARG	1.94E-14		45
MYCN	2.51E-13		32
Top Molecular and Cellular Functions	*p*-value		# Genes
Cellular Growth and Proliferation	1.96E-04 - 1.11E-16		285
Cellular Movement	2.13E-04 - 3.09E-14		171
Lipid Metabolism	2.79E-04 - 7.14E-13		129
Molecular Transport	2.72E-04 - 7.14E-13		176
Small Molecule Biochemistry	2.79E-04 - 7.14E-13		163
Physiological System Development and Function	*p*-value		# Genes
Cardiovascular System	2.58E-04 - 7.81E-12		107
Organismal Development	2.58E-04 - 7.81E-12		181
Organismal Survival	4.90E-05 - 3.31E-10		175
Hematological System	2.72E-04 - 3.05E-08		123
Immune Cell Trafficking	2.72E-04 - 3.05E-08		72
Top Up-regulated genes	HG/LG Ratio	Top Down-regulated genes	HG/LG Ratio
*FN1*	12.58	*TGM2*	−5.86
*SCD*	10.69	*MED27*	−5.06
*PPP1R9A*	9.23	*SESTD1*	−4.07
*XYLT2*	5.14	*ALB*	−3.80
*PIGC*	4.52	*RPL18A*	−3.78
*NFASC*	4.02	*MT-ND1*	−3.21
*PLEC*	3.55	*HSD11B1*	−3.11
*ABHD3*	3.40	*BM2*	−3.00
*FASN*	3.34	*TAGLN*	−2.95
*ARID4B*	3.30	*ANGPTL3*	−2.94

Ingenuity Pathway Analysis (IPA) was used for functional annotation and mapping of 647 DE genes from the time-course microarray analysis of abdominal fat in HG and LG cockerels (1-11 wk) that were “Analysis Ready” (AR-DE). This unique (non-redundant) gene set was compiled from AR-DE genes found in the main effect of genotype or the genotype x age interaction

Annotated lists of AR-DE genes are provided in eight worksheets of Additional file 6, which show top canonical pathways and biological functions [“Upstream Regulators (22 AR-DE genes), Oxidative Phosphorylation (19 AR-DE genes), LXR-RXR Activation (9 AR-DE genes), Fatty Acid Metabolism (61 AR-DE genes), Adipogenesis (12 AR-DE genes), Insulin Resistance (27 AR-DE genes), VEGF Signaling (8 AR-DE genes), and Protein Metabolism” (44 AR-DE genes)] revealed by time-course microarray analysis. Fourteen of the 22 AR-DE transcription factors identified by microarray analysis are expressed higher in abdominal fat of the LG cockerels (1-11 wk). Likewise, 15 of the 19 AR-DE genes involved in oxidative phosphorylation are more abundant in the LG. Of the nine AR-DE genes belonging to the LXR-RXR pathway, only three genes encoding transport proteins (*TF, APOA1* and *ALB*) are up-regulated in visceral fat of the LG birds.

#### Gene interaction networks identified from the time-course microarray analysis (1-11 wk)

**Fig. 3:**
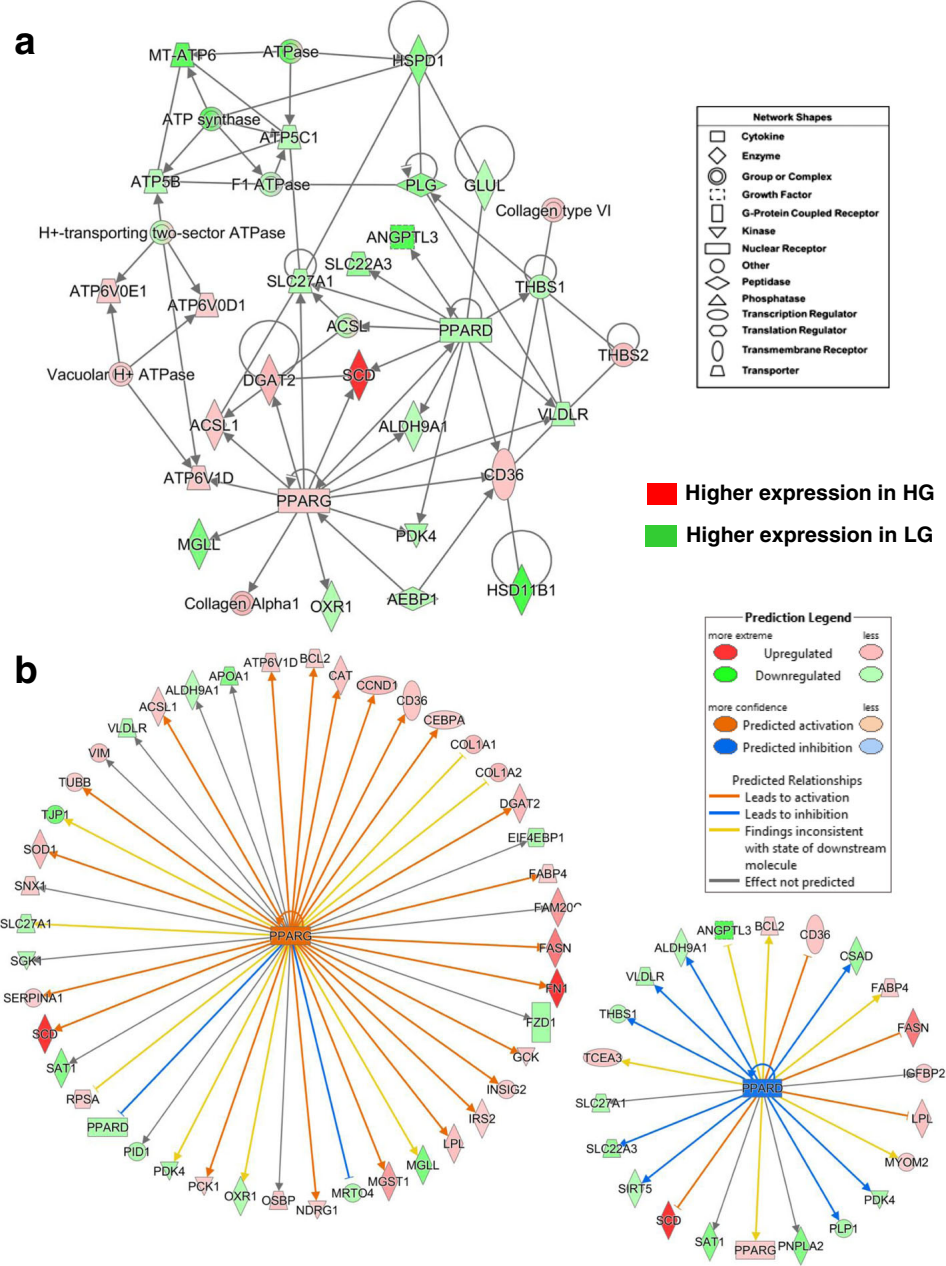
Interactions of two transcription factors (PPARG and PPARD) and their direct target genes that control lipid metabolism in abdominal fat of juvenile HG and LG chickens. This gene interaction network (**a**) was identified by microarray analysis and Ingenuity® Pathway Analysis (IPA) from a combined unique list (647 AR-DE genes) merged from DE genes in the main effect of genotype (averaged across 6 ages) with DE genes from the interaction of age and genotype (see Fig. 2). Red gene symbols indicate higher expression in abdominal fat of HG birds, while green gene symbols indicate higher expression in the LG. Ingenuity® Upstream Regulator Analysis identified direct target genes (**b**) of two opposing ligand-activated transcription factors (*PPARG* and *PPARD*). IPA predicts the activation of PPARG (orange symbol and arrows) and inhibition of PPARD (blue symbols and arrows), based on the observed expression of DE genes and the expected mammalian responses found in the Ingenuity Knowledge Base

A top gene interaction network (Fig. 3-a), identified by IPA as “Lipid Metabolism”, shows interaction of two opposing transcription factors [peroxisome proliferator-activated receptor gamma (*PPARG*) and peroxisome proliferator-activated receptor delta (*PPARD*)] with their respective direct target genes. Six direct targets of the *PPARG* are metabolic enzymes up-regulated in abdominal fat of the HG chickens [stearoyl-CoA desaturase (delta-9-desaturase; *SCD*); diacylglycerol O-acyltransferase 2 (*DGAT2*), acyl-CoA synthetase long-chain family member 1 (*ACSL1*), (ATPase, H^+^-transporting, lysosomal 34 kDa, V1 subunit D *(ATP6V1D*), collagen alpha 1 (*COL1A1*), and thrombospondin receptor (*CD36*)]. The expression of several other genes were downregulated in the HG cockerels (or expressed higher in LG) [*PPARD*, *ALDH9A1,* monoglyceride lipase (*MGLL*), oxidation resistance 1 (*OXR1*), and pyruvate dehydrogenase kinase, isozyme 4 (*PDK4*)]. The adipocyte enhancer binding protein 1 (*AEBP1*), which was expressed higher in abdominal fat of LG birds, directly affects both *PPARG* and *CD36*. On the other hand, *PPARD* and many of its direct target genes (10 AR-DE genes) are up-regulated in the slower-growing and leaner LG genotype, with the exception of two genes (*SCD* and *CD36*), which were highly expressed in the HG. The two opposing transcription factors (*PPARG* and *PPARD*) also share several down-regulated target genes in the HG, including *PDK4, ALDH9A1, ACSL, SLC27A1* and *VLDLR*. The potent LPL inhibitor, angiopoietin-like 3 (*ANGPTL3*), solute carrier family 22 (organic cation transporter), member 3 (*SLC22A3*), glutamate-ammonia ligase (*GLUL*), and thrombospondin 1 (*THBS1*), like *PPARD*, are up-regulated in abdominal fat of LG cockerels. Additional genes with higher expression in the LG include plasminogen (*PLG*), several ATPases, heat shock 60 kDa protein 1 or chaperonin (*HSPD1*) and hydroxysteroid (11-beta) dehydrogenase 1 (*HSD11B1*), which degrades glucocorticoid, interact with direct targets of PPARD (i.e., *VLDLR, THBS1* and *CD36*), whereas, *THBS2* and *collagen type VI* were up-regulated in visceral fat of the HG chickens*.* Interestingly, an equal number of unique ATPases were associated with either PPARG in the HG or PPARD in the LG chickens.

Numerous direct targets of PPARG were identified by Ingenuity Upstream Regulator Analysis from DE genes identified by the time-course (1-11 wk) microarray study of abdominal fat of HG and LG chickens (Fig. 3-b). Twenty-seven direct DE genes of PPARG were expressed at higher levels in abdominal fat of the HG chickens, whereas 13 direct gene targets were more abundant in the LG chickens. This detailed view of PPARG target genes predicts (based on literature accrued in the Ingenuity Knowledge Base and observed expression values) that PPARG up-regulates or activates 31 direct gene targets (orange arrows) in the HG chickens. Among these up-regulated genes, most control lipogenesis, adiposity and energy metabolism (*SCD*, *FASN, FN1, DGAT2, CEBPA, LPL, FABP4, INSIG2, IRS1, PCK1, SCD, SOD1, SERPINA1,* and *ACSL1*). Whereas, *PPARD* expression was higher in the LG chickens and predicted to be inhibited (blue color arrows) and downregulated in the HG chickens (or higher in the LG), which agrees with our observed expression values (Fig. 3-b). Eight down-regulated genes (*PDK4, PLP1, SIRT5, SLC22A3, THBS1, VLDLR, ALDH9A1, ANGPTL3,* and *CSAD*) were predicted to be inhibited by PPARD (indicated by the blue arrows) in visceral fat of the HG; these genes were actually expressed higher in the slow-growing LG chickens as indicated by the green-colored gene symbols.

**Fig. 4:**
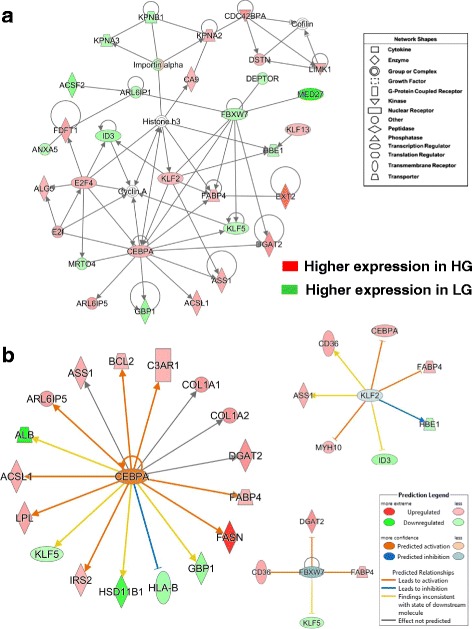
This adipogenic gene network is centered on the up-regulated transcription factor *CEBPA* and interactions with several other transcription regulators (**a**). Another major gene interaction network identified by a time-course microarray analysis of abdominal fat of HG and LG chickens (1-11 wk). Ingenuity Pathway Analysis (IPA) identified this functional gene interaction network from the unique set of 647 AR-DE genes shown in the Venn diagram (Fig. 2a). Red gene symbols indicate higher expression in HG adipose tissue, while green gene symbols indicate higher expression in visceral fat of the LG. The Ingenuity® Upstream Regulator Analysis determined additional direct target genes of CEBPA, KLF2 and FBXW7 (**b**). Ingenuity predicts the activation of *CEPBA* *(orange* gene symbol and arrows) and inhibition of *KLF2* and *FBXW7* (*blue* symbol and *arrow*)

Another gene interaction network controlling lipid metabolism (Fig. 4-a), centered on the up-regulated transcription factor CCAAT/enhancer binding protein, alpha (*CEBPA*), was found in a subset of 252 commonly shared genes from the microarray analysis (see Fig. 2-a). CEBPA directly interacts with seven up-regulated genes in HG birds [argininosuccinate synthase 1 (*ASS1*)*,* acyl-CoA synthetase long-chain family member 1 (*ACSL1), DGAT2, FABP4,* Kruppel-like factor 2 (*KLF2*)*,* E2F transcription factor 4 (*E2F4*), and ADP-ribosylation factor-like 6 interacting protein 5 (*ARL6IP5*)]. Four direct targets of CEBPA were down-regulated in HG abdominal fat including F-box and WD repeat domain containing 7, E3 ubiquitin protein ligase (*FBXW7*), Kruppel-like factor 5 (*KLF5*), guanylate binding protein 1, interferon-inducible (*GBP1*), and mRNA turnover 4 homolog, *S. cerevisiae* (*MRTO4*). Ubiquitin ligase (*FBXW7*) has direct interactions with *DGAT2, FABP4, KLF2*, *KLF5,* ADP-ribosylation factor-like 6 interacting protein 1 (*ARL6IP1*), DEP domain containing MTOR-interacting protein (*DEPTOR*) and mediator complex subunit 27 (*MED27*). Other members of this lipogenic network include exostosin glycosyltransferase 2 (*EXT2*), hemoglobin, epsilon 1 (*HBE1*), Kruppel-like factor 13 (*KLF13*), carbonic anhydrase IX (*CA9*), inhibitor of DNA binding 3 (*ID3*), dolichyl-phosphate beta-glucosyltransferase (*ALG5*), annexin A5 (*ANXA5*), farnesyl-diphosphate farnesyltransferase 1 (*FDFT1*) and acyl-CoA synthetase family member 2 (*ACSF2*).

Ingenuity Upstream Regulator Analysis identified 18 AR-DE genes as direct targets of CEPBA (Fig. 4-b), 13 lipogenic AR-DE genes were up-regulated in the HG genotype (*FASN, DAGT2, LPL, CEBPA, FABP4* and *IRS2*). Whereas, only five AR-DE genes were downregulated in the HG birds (*ALB*, *KLF5*, *HSD11B1, HLA-B,* and *GBP1*) in the subset of 252 commonly-shared genes from microarray contrasts (see Fig. 2-a). The Ingenuity Upstream Regulator Analysis predicts that CEBPA was activated in the HG chickens and led to activation of eight genes (orange arrows) or inhibition (blue blunted line) of a single gene, major histocompatibility complex, class I, B (*HLA-B*). Based on our observed expression values and expected responses from the mammalian-based Ingenuity Knowledge Base, the Upstream Analysis predicts that *KLF2* would be inhibited (blue gene symbol), while clearly its expression was more abundant in HG abdominal fat. *KLF2* was predicted to inhibit three major genes (*CEBPA, FABP4*, and *MYH10*), highly expressed in the HG, and would further inhibit the embryonic form of hemoglobin, epsilon 1 (*HBE1*). The yellow arrows point to a contradiction between information accrued in the Ingenuity Knowledge Base and the expected state (inhibited, blue) of two target DE genes [thrombospondin receptor (*CD36*) and *ASS1*], although their actual state was upregulated (activated, red symbol). There is predicted uncertainty (grey arrows) about *KLF2* inhibition of ID3 (blunted-yellow line), which is down regulated in the HG. Ingenuity correctly predicts *FBXW7* to be inhibited (blue symbol), since it was expressed lower in HG, and that this ubiquitin protein ligase would directly block up-regulation of three ligogenic genes (*CD36*, *DGAT2* and *FABP4*) (blunt orange line), along with uncertainty about the ability of *FBXW7* to block (blunt yellow line) the down-regulated *KLF5* (green symbol). It is clear from these observations that CEBPA is major lipogenic transcription factor and a major contributor to the higher growth and greater fat accretion found in the HG cockerels.

#### RNA-Seq analyses of abdominal fat in four HG and four LG chickens at 7 wk

RNA-Seq analysis revealed 2410 DE (FDR≤0.05; FPKM > 0.5) genes at 7 wk. (Additional file 5), which was the age of greatest extremes in growth and fatness phenotypes exhibited by HG and LG cockerels. IPA identified 2026 DE genes from the RNA-Seq dataset as annotated and “Analysis Ready” (AR) from the RNA-Seq dataset. A summary of the IPA of this RNA-Seq dataset is presented in Table 3. The top five canonical pathways populated by DE genes from the RNA-Seq analysis were “Axonal Guidance Signaling, Acute Phase Response Signaling, Role of Tissue Factor in Cancer, Coagulation System and Complement System” (see Additional file 7 for detailed gene lists). The top five “Upstream Regulators” predicted by Ingenuity Upstream Analysis were ESR1 (234 AR-DE targets), TP53 (230 AR-DE targets), CTNNB1 (132 AR-DE targets), ESR2 (83 AR-DE targets), and SMARCA4 (105 AR-DE targets). The top “Molecular and Cellular Functions” over-represented by DE genes from the RNA-Seq analysis were “Cellular Movement” (538 AR-DE genes), “Cellular Growth and Proliferation” (866 AR-DE genes), “Cellular Morphology” (606 AR-DE genes), “Cell Death and Survival” (706 AR-DE genes), and “Cellular Assembly and Organization” (452 AR-DE genes). The top “Physiological System Development and Function” categories represented by RNA-Seq DE genes were “Organismal Development” (701 AR-DE genes), “Tissue Development” (801 AR-DE), “Cardiovascular System” (373 AR-DE genes), “Embryonic Development” (476 AR-DE genes) and “Organismal Survival” (530 AR-DE genes). The top ten up-regulated genes in HG abdominal fat included *SCD,* whereas the top ten up-regulated genes in the LG cockerels mainly encode hemostasis (*FGA, FGB, FGG, PLG* and *KNG1*) and transport or binding (*ALB, GC, APOH* and *FABP1*) proteins.

**Table 3:** IPA summary of RNA-Seq analysis of abdominal fat in HG and LG cockerels (7 wk)

Top Canonical Pathways	*p*-value	Overlap	Ratio
Axonal Guidance Signaling	1.07E-11	20.3%	88/434
Acute Phase Response Signaling	4.80E-11	27.2%	46/169
Role of Tissue Factor in Cancer	4.05E-10	30.9%	34/110
Coagulation System	4.13E-09	48.6%	17/35
Complement System	1.20E-08	45.9%	17/37
Top Upstream Regulators	*p*-value of overlap		#Target Genes
ESR1	6.70E-29		234
TP53	1.48E-24		230
CTNNB1	4.21E-19		132
ESR2	1.14E-17		83
SMARCA4	3.03E-13		105
Top Molecular and Cellular Functions	*p*-value		# Genes
Cellular Movement	7.08E-08 – 5.98E-43		538
Cellular Growth and Proliferation	7.49E-08 - 3.56E-37		866
Cell Morphology	1.37E-07 - 5.49E-35		606
Cell Death and Survival	1.31E-07 - 2.52E-30		706
Cellular Assembly and Organization	3.03E-08 - 2.15E-29		452
Physiological System Development and Function	*p*-value		# Genes
Organismal Development	1.40E-07 - 1.18E-29		701
Tissue Development	1.53E-07 - 9.90E-28		801
Cardiovascular System	1.47E-07 - 5.03E-27		373
Embryonic Development	5.07E-08 – 7.56E-25		476
Organismal Survival	8.82E-09 - 1.54E-24		530
Top Up-regulated Genes	Log2 Ratio	Top Down-regulated Genes	Log2 Ratio
*MYL1*	7.55	*GC*	−8.52
*SCD*	7.02	*FGB*	−8.34
*ACTN2*	6.15	*PLG*	−8.23
*CASQ2*	5.91	*AHSG*	−8.18
*TNNC2*	5.72	*FGA*	−8.14
*MYH3*	5.58	*HRG*	−7.73
*RIPPLY3*	5.03	*FGG*	−7.62
*TNNI2*	4.44	*ALB*	−7.40
*SMOC1*	4.13	*APOH*	−6.93
*ABHD3*	4.08	*KNG1*	−6.63

Ingenuity Pathway Analysis (IPA) was used for functional annotation and mapping of 2026 “Analysis Ready” DE genes identified by RNA-Seq analysis of abdominal fat in four HG and four LG cockerels at 7 wk. These tissue samples were from the same HG and LG birds used for microarray analysis at this age

Additional functional categories and canonical pathways represented by AR-DE genes from the RNA-Seq analysis (Additional file 7) include “Upstream Regulators (58 genes), Acute Phase Signaling (48 genes, Coagulation System (17 genes), Intrinsic Prothrombin Activation (12 genes), Extrinsic Prothrombin Activation (11 genes), LXR/RXR Activation (31 genes), Fatty Acid Metabolism (158 genes), Adipogenesis Pathway (26 genes), Insulin Resistance (71 genes), VEGF Signaling (18 genes), and Protein Metabolism” (63 genes). Ingenuity Analysis recognized 58 AR-DE genes as “Upstream Regulators”, where 24 AR-DE transcription factors were expressed higher in HG abdominal fat and 34 AR-DE upstream regulators were up-regulated in the LG. The “Acute Phase Signaling Pathway” was also overpopulated by 40 AR-DE genes that were up-regulated in the LG, whereas, only 8 genes in this pathway were expressed in abdominal fat of the HG. Likewise, only a single up-regulated gene in the HG was assigned to the canonical “Coagulation System” (*PLAU*) and “Intrinsic Prothrombin Activation Pathway” (*COL18A1*), while 11 AR-DE genes belonging to the “Extrinsic Prothrombin Activation Pathway” were all over-expressed in the LG. Similarly, the “LXR-RXR Activation Pathway” was composed of 23 AR-DE genes highly expressed in LG visceral fat compared to only 8 up-regulated genes from the HG birds. A total of 158 AR-DE genes were assigned by IPA to “Fatty Acid Metabolism” and of these 84 AR-DE genes were up-regulated in the LG cockerels compared to 76 genes expressed high in the HG. Another critical canonical pathway identified was “Adipogenesis”, which was populated by 15 up-regulated AR-DE genes in the LG and 11 up-regulated genes in the HG cockerels. Insulin resistance is another important biological process related to visceral fatness and endocrine signaling, where 41 AR-DE genes were expressed higher in the LG chickens and 30 genes were more abundant in the HG. The vascular endothelial growth factor (VEGF) signaling pathway was dominated by 13 up-regulated AR-DE genes in the HG birds, with only 5 genes up-regulated in the LG. In contrast, protein metabolism had an equal number of AR-DE genes from HG (31 up-regulated genes) and LG (32 up-regulated genes) cockerels at 7 wk.

#### Gene interaction networks revealed from RNA-Seq analysis of HG and LG abdominal fat

**Fig. 5:**
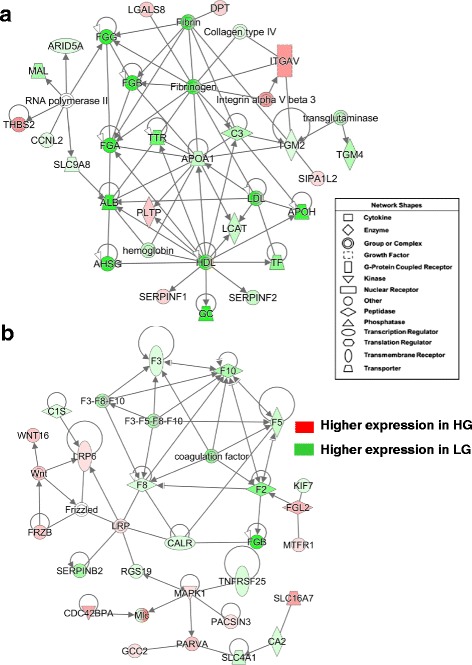
RNA-Seq analysis of abdominal fat at 7 wk identified two gene interaction networks controlling fibrinogenesis and hemostasis that are populated by genes highly expressed in LG cockerels. Panel **a** shows an interaction network functionally annotated by IPA as “Cell-to-Cell Signaling, Hematological System Development and Function”. The second panel (**b**) shows a gene network, annotated by IPA as “Hematological Disease”, which involves interaction of a large cluster of clotting factors with multiple cell signaling components

From 2410 DE genes identified by RNA-Seq analysis of abdominal fat in HG and LG cockerels at 7 wk, a total of 2026 DE genes were determined by IPA as “Analysis Ready” (AR) and subjected to an IPA “Core Analysis”. Two gene interaction networks were highly populated by several hemostasis genes that were highly expressed in abdominal fat of the LG chickens at 7 wk (Fig. 5). Panel A shows a gene interaction network functionally annotated by IPA as “Cell-to-Cell Signaling, Hematological System Development and Function”. This network was composed of several DE genes involved in acute phase signaling and blood coagulation (see Additional file 7). These genes included fibrinogen A, B and G (*FGA, FGB* and *FGG*), molecular transporters [albumen (*ALB*), transthyretin (*TTR*), apolipoprotein A-I (*APOA1*), apolipoprotein H (*APOH*), transferrin (*TF*), group-specific component (*GC*; Vitamin D binding protein), solute carrier family 9, subfamily A (*SLC9A8*), serpin peptidase inhibitor, clade F, member 2 (*SERPINF2*), and alpha-2-Heremans-Schmid-glycoprotein (*AHSG* or *fetuin-A*). Other genes expressed higher in visceral fat of the LG chickens include lecithin-cholesterol acyltransferase (LCAT), complement component 3 (*C3*), transglutaminase 2 and 4 (*TGM2* and *TGM4*), mal or T-cell differentiation protein (*MAL*), cyclin L2 (*CCNL2*), and AT rich interactive domain 5A (*ARID5A*). Other genes expressed higher in the HG chickens were dermatopontin (*DPT*), thrombospondin 2 (*THBS2*), serpin peptidase inhibitor, clade F, member 1 (*SERPINF1*), which is a neurotrophic factor and potent inhibitor of angiogenesis, phospholipid transfer protein (*PLTP*), integrin, alpha V (*ITGAV*), and lectin, galactoside-binding, soluble, 8 (*LGALS8*).

The other hemostatic gene network revealed by RNA-Seq analysis (Fig. 5-b) was composed of several coagulation factors (*F2*, *F3, F5, F8, F10, SERPINB2,* and *FGB*), which were all over-expressed in abdominal fat of LG cockerels. The complement component (*C1S*), calreticulin (*CALR*), the anion exchanger *SLC4A1*, kinesin family member 7 (*KIF7*), carbonic anhydrase II (*CA2*) and regulator of G-protein signaling 19 (*RGS19*) were also expressed higher in the LG. Several additional genes in this direct interaction network were up-regulated in the HG, including mitogen-activated protein kinase 1 (*MAPK1*), parvin alpha (*PARVA*), the monocarboxylate transporter *SCL16A7*, coiled-coil domain containing 2 (*GCC2*), CDC42 binding protein kinase alpha (*CDC42BPA*), LDL receptor related protein 6 (*LRP6*), frizzled-related protein (*FRZB*), wingless-type MMTV integration site family member 16 (*WNT16*), protein kinase C and casein kinase substrate in neurons 3 (*PACSIN3*), fibrinogen-like 2 (*FGL2*) and mitochondrial fission regulator 1 (*MTFR1*). This gene interaction network was functionally annotated by IPA as “Hematological Disease”.

**Fig. 6:**
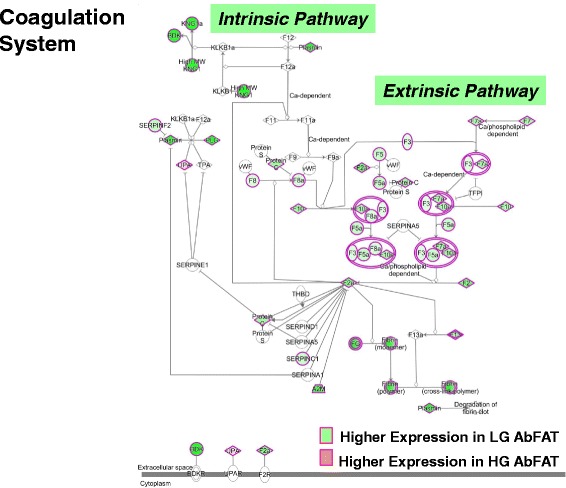
Ingenuity Pathway Analysis of RNA-Seq data showed that the canonical coagulation system was over-represented by up-regulated genes in abdominal fat of LG cockerels. Plasminogen activator, urokinase (*UPA*) was the only coagulation gene expressed higher in visceral fat of the HG birds. The IPA indicates that 16 out of 17 DE genes are expressed at higher levels in the LG birds, while only plasmogenin activator, urokinase was up-regulated in abdominal fat of HG cockerels. Both the intrinsic (12 AR-DE genes) and extrinsic (11 AR-DE genes) pathways in visceral fat of LG birds are highly-populated by up-regulated coagulation genes (see Additional file 7 for functional AR-DE gene lists)

Sixteen of the 17 DE genes were over expressed in the IPA canonical coagulation system, which includes the extrinsic and intrinsic prothrombin activation pathways (Fig. 6; Additional file 7). The up-regulated hemostasis genes found in the LG chickens include six coagulation factors (*F2, F3, F5, F8, F10* and *F13B*), three fibrinogen subunits (*FGA, FGB* and *FGG*), two serpin peptidase inhibitors (*SERPINC1* and *SERPINF2*), plasminogen (*PLG*), kininogen 1 (*KNG1*), bradykinin (*BDK*), protein C (*PROC*), and von Willebrand factor, C and EGF domains (*VWCE*). In contrast, plasminogen activator, urokinase (*PLAU*) was the only coagulation-related gene that was expressed higher in HG abdominal fat. Clearly, the diminished abdominal fat mass of slower-growing LG cockerels reflects a highly prothrombotic state; although the consequence on endocrine signaling and/or lipid metabolism remains unknown. However, microarray analysis of liver in the HG and LG cockerels does show higher expression of four coagulation genes (*FGA, FGB, SERPINF2*, and *KNG1*) in the LG birds (Cogburn, LA unpublished data). Presently, we do not know if local activation of the coagulation system in adipose tissue (or liver) affects the systemic hemostatic mechanism, which normally would rely on availability of coagulation precursors synthetized mainly in the liver.

**Fig. 7:**
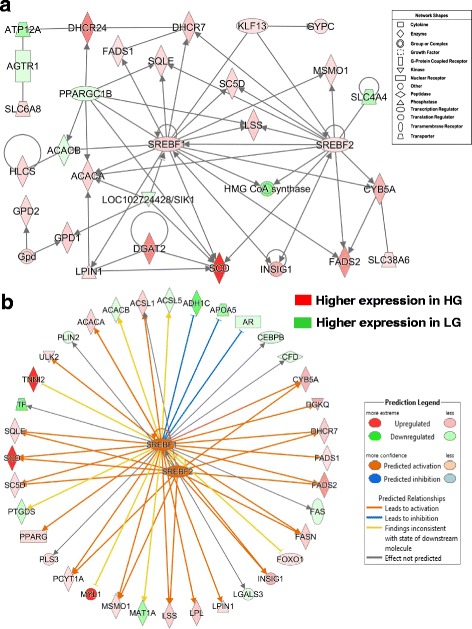
IPA of RNA-Seq data revealed a gene network controlled by the lipogenic transcription factors *SREBF1* and *SREBF2* (**a**). Panel **b** shows additional direct gene targets that are either unique to, or shared by, *SREBF1* (35 DE genes) and *SREBF2* (16 DE genes). The Ingenuity® Upstream Analysis predicates activation (*orange* arrows and symbols) or inhibition (blunt blue line) of direct target genes. The predicted activated (*orange arrows*) or inhibited (blunt blue lines) state of DE target genes is based on the expected responses accrued in the Ingenuity Knowledge Base

One metabolic gene network, annotated by IPA as “Lipid Metabolism, Small Molecule Biochemistry and Energy Production”, was centered on interactions of two transcription factors (*SREBF1* and *SREBF2*) and their numerous activated target genes, most of which were highly expressed in abdominal fat of the faster-growing and fatter HG cockerels at 7 wk (Fig. 7-a). The two highest-expressed genes found in this lipogeneic network were *SCD* (10.7-fold higher) and 24-dehydrocholesterol reductase (*DHCR24*) [5.5-fold higher], the final enzyme in cholesterol biosynthesis. Other up-regulated genes in visceral fat of HG chickens were diacylglycerol O-acyltransferase 2 (*DGAT2*), lipin 1 (*LPIN1*), insulin induced gene 1 (*INSIG1*), *ACACA, FADS1, SQLE, SC5D, LSS, FADS2, CYB5A, SLC38A6, MSMO1, KLF13* and glycophorin C (*GYPC*). Only seven genes in this network were expressed higher in abdominal fat of the LG cockerels [*AGTR1, ATP12A,* PPARG coactivator 1 beta (*PPARGC1B*)*, ACACB*, salt inducible kinase 1 (*SIK1*), HMG CoA synthase (*HMGCS*) and solute carrier family 4 member 4 (*SLC4A4*).

Ingenuity® Upstream Regulator Analysis identified 36 AR-DE genes that are direct targets of *SREBF1* (22 genes were expressed higher in the HG, which is consistent with activated *SREBF1*) and 16 AR-DE genes that are direct targets of *SREBF2* (12 genes were expressed higher in the HG, which is consistent with activation of *SREBF2*) (Fig. 7-b). As direct targets of *SREBF1*, the four most abundant genes in abdominal fat of the HG were myosin light chain 1 (*MYL1*), SCD, troponin I type 2, skeletal, fast (*TNNI2*) and *FADS2,* whereas *MAT1A, APOA5, TF* and *ADH1C* were the highest-expressed target genes of *SREBF1* found in the LG at 7 wk. The Ingenuity Upstream Analysis predicts that both *SREBF1* and *SREBF2* are activated (orange gene symbols) and that 19 DE target genes would be activated (orange arrows). Twelve of these highly-expressed genes are activated by both transcription factors (*ACSL1, CYB5A, DHCR7, FADS2, FASN, INSIG1, LSS, MSMO1, PCYT1A, SC5D, SCD* and *SQLE*). *SREBF1* appears to inhibit only three genes [alcohol dehydrogenase 1C (class I), gamma polypeptide (*ADH1C*), apolipoprotein A-V (*APOA5*) and androgen receptor (*AR*)], which were over-expressed in the LG chickens*.* Only 13 direct target genes of *SREBF1* and/or *SREBF2* were expressed higher in abdominal fat of the LG chickens. Perilipin 2 (*PLIN2*) was the only direct target of *SREBF2* that was expressed higher in visceral fat of the LG cockerels.

**Fig. 8:**
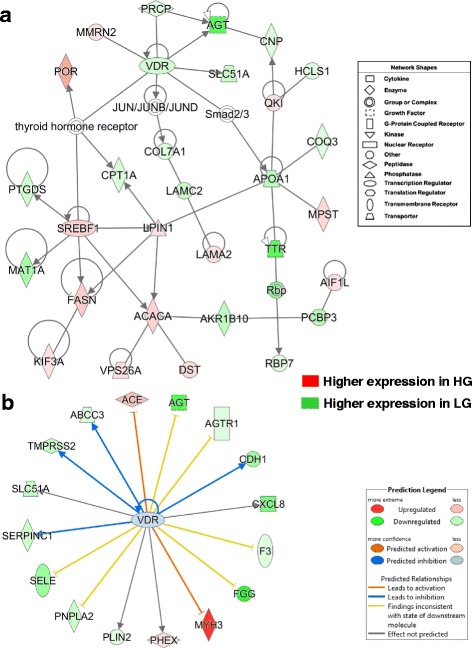
Gene network showing interactions of two ligand-activated transcription factors (*VDR* and *SREBF1*) from RNA-Seq analysis of abdominal fat in HG and LG cockerels at 7 wk. (**a**). This network of 30 DE genes was functionally annotated by IPA as “Energy Production; Lipid Metabolism”. The expression of 13 DE genes was higher in abdominal fat of HG cockerels, while 17 DE genes were more abundant in the LG. Panel **b** provides 16 direct targets of VDR, and *VDR* itself, which were largely up-regulated in LG abdominal fat at 7 wk., only three genes were expressed higher in the HG. Upstream Regulator Analysis predicted inhibition of the *VDR* gene (blue symbol) and inhibition (*blue* arrow or blunt lines) of target genes reflecting its up-regulation in the LG

RNA-Seq analysis revealed interactions between the vitamin D receptor (*VDR*) and several direct targets of *SREBF1* including *FASN, ACACA* and *LPIN1*, which were up-regulated in the HG birds (Fig. 8-a). Other up-regulated genes in the HG chickens include cytochrome p450 oxidoreductase (POR), multimerin 2 (*MMRN2*), quaking *(QKI),* mercaptopyruvate sulfurtransferase (*MPST*), laminin subunit alpha 2 (*LAMA2*), allograft inflammatory factor 1 like (*AIF1L*), dystonin (*DST*), VPS26 retromer complex component A (*VPS26A*) and kinesin family member 3A (*KIF3A*). Another group of 15 genes were up-regulated in abdominal fat of the LG [*APOA1, TTR, RBP7,* angiotensinogen (*AGT*)*,* aldo-keto reductase family 1, member B10 (*AKR1B10*), poly(rC) binding protein 3 (*PCBP3*), coenzyme Q3, methyltransferase (*COQ3*), laminin subunit gamma 2 (*LAMC2*), collagen type VII alpha 1 (*COL7A1*), hematopoietic cell-specific Lyn substrate 1 (*HCLS1*), 2′,3′-cyclic nucleotide 3′ phosphodiesterase (*CNP*), prolylcarboxypeptidase (*PRCP*), methionine adenosyltransferase 1A (*MAT1A*), prostaglandin D2 synthase (*PTGDS),*carnitine palmitoyltransferase 1A (*CPT1A*), and solute carrier family 51 alpha subunit (*SLC51A*)]*.*

Ingenuity Upstream Regulator Analysis predicts that the expression of VDR (Fig. 8-b) would be inhibited (blue gene symbol), which would lead to down-regulation of four direct target genes [serpin peptidase inhibitor, clade C (antithrombin), member 1 (*SERPINC1*), transmembrane protease, serine 2 (*TMPRSS2*), ATP binding cassette subfamily C member 3 (*ABCC3*), and cadherin 1 (*CDH1*). Of the 17 DE direct targets of the VDR, three genes [angiotensinogen converting enzyme (*ACE*), myosin, heavy chain 3, skeletal muscle, embryonic (*MYH3*) and phosphate regulating endopeptidase homolog, X-linked (*PHEX*)] were expressed at higher levels in abdominal fat of the HG chickens. The up-regulated expression of six genes in the LG birds (*AGT, AGTR1, F3, FGG, PNPLA2* and *SELE*) was inconsistent with the expected state of these genes (blunted yellow edges) from the Ingenuity Knowledge Base. However, the effect of the VDR was not predicted for three AR-DE genes [the inflammatory chemokine, C-X-C motif chemokine ligand 8 (*CXCL8*), phosphate regulating endopeptidase homolog, X-linked (*PHEX*) and perilipin 2 (*PLIN2*).

**Fig. 9:**
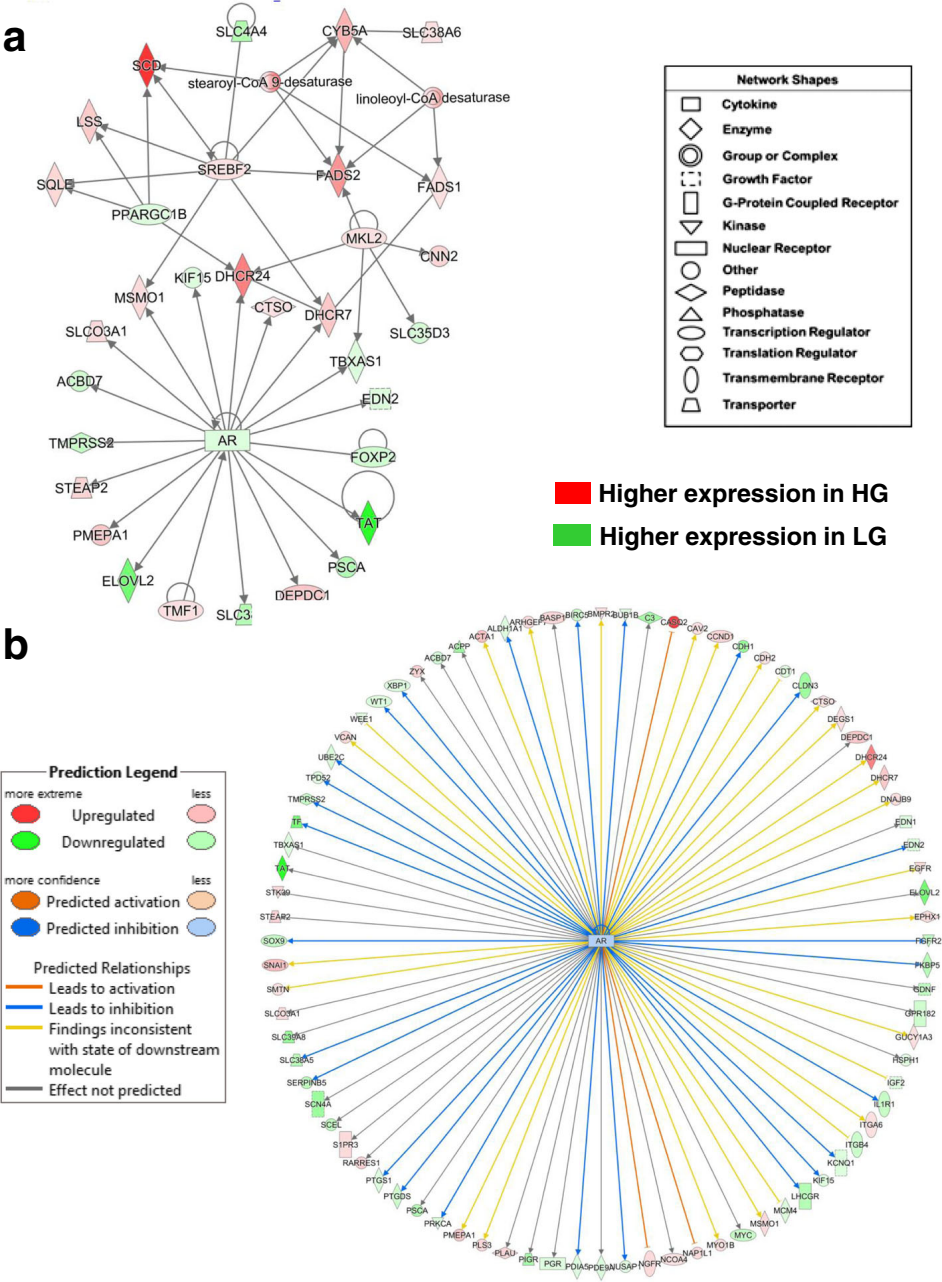
RNA-Seq analysis of abdominal fat in HG and LG cockerels at 7 wk. shows a gene interaction network centered on interactions between *SREBP2* and the *AR* (**a**). *SREBP2* and its direct targets are expressed higher in the HG cockerels, while the *AR* and 8 target genes were up-regulated in the LG. Ingenuity Upstream Analysis predicts that the *AR* should be activated in the HG (orange gene symbol), based on the up-regulated condition (orange arrows and red gene symbols) of 27 target genes (**b**). An additional 22 DE genes, known direct targets of the AR, were up-regulated (green gene symbols) in abdominal fat of the LG birds. Ingenuity predicted activation is indicated by *orange* arrows), while predicted inhibition is shown by blunted *blue* lines

Another gene network functionally annotated by IPA as controlling lipid metabolism was centered on the androgen receptor (*AR*), which was expressed higher in abdominal fat of the LG cockerels, and the interaction of its direct target genes with three additional transcription factors [SREBF2, PPARG coactivator 1 beta (*PPARGC1B*) and MKL1/myocardin like 2 (*MKL2*)] (Fig. 9-a). Eight genes are direct targets of the lipogenic transcription factor *SREBF2*, where *CYB5A*, *FADS2, DHCR7, MSMO1, SQLE, LSS* and *SCD* were up-regulated in HG visceral fat, and only solute carrier family 4 member 4 (*SLC4A4*) was expressed higher in the LG. Two direct targets of the AR [methylsterol monooxygenase 1 (*MSMO1*) and 7-dehydrocholesterol reductase (*DHCR7*)] are also direct targets of SREBF2, while *DHCR24* is a direct target of the *AR* and *PPARGC1B,* both of these transcription regulators were up-regulated in LG chickens. *PPARGC1B* also shares three genes that are direct targets of *SREBF2* [i.e., squalene epoxidase (*SQLE*), lanosterol synthase (*LSS*) and *SCD*]. Three additional targets of *MKL2* are solute carrier family 35 member D3 (*SLC35D3*), a key regulator of dopamine signaling, and thromboxane A synthase 1 (*TXBAS1*) both of which were up-regulated in abdominal fat of the LG cockerels, whereas calponin 2 (*CNN2*) is a third target gene of *MKL2*, which was expressed higher in the HG chickens. Fourteen additional genes in this network are direct targets of the AR, where 9 genes are expressed higher in the LG and 5 up-regulated in the HG. Among the 9 upregulated target genes of AR found in visceral fat of the LG line were endothelin 2 (*EDN2*), forkhead box P2 (*FOXP2*), tyrosine aminotransferase (*TAT*), prostate stem cell antigen (*PSCA*), the sodium-coupled amino acid transporter (*SLC38A5*), ELOVL fatty acid elongase 2 (*ELOVL2*), transmembrane protease, serine 2 (*TMPRSS2*), acyl-CoA binding domain containing 7 (*ACBD7*) and kinesin family member 15 (*KIF15*). Ingenuity Upstream Regulator Analysis identified 85 direct targets of the AR gene from the RNA-Seq analysis of abdominal fat in the HG and LG cockerels at 7 wk. (Fig. 9-b). Thirty-five of the known direct targets of the AR were upregulated in HG cockerels, while 50 DE genes were over expressed in visceral fat of the LG birds. The predicted inhibition of the AR and 26 of its target genes is based on the observed down-regulation of the *AR* (blue symbol and arrows) in the HG abdominal fat (or up-regulation in LG birds as indicated by green gene symbols) from the RNA-Seq analysis. The two highest up-regulated DE genes in the HG among direct targets of the AR were calsequestrin 2 (*CASQ2*) and 24-dehydrocholesterol reductase (*DHCR24*), which catalyzes the final step in cholesterol synthesis. Another terminal enzyme of cholesterol synthesis, *DHCR7,* was also upregulated in abdominal fat of the HG cockerels. Ingenuity Upstream Analysis indicated that three up-regulated genes in the HG cockerels [*CASQ2,* nucleosome assembly protein 1 like 1 (*NAP1L1*), and nerve growth factor receptor (*NGFR*)] would be actively blocked (red blunted lines) by the AR. Another steroid hormone receptor up-regulated in visceral fat of the LG cockerels was the progesterone receptor (*PGR*), which also had a large number of direct target genes (36 were upregulated in the LG and 25 DE genes were expressed higher in the HG birds) in the RNA-Seq dataset.

### Verification of differential gene expression by quantitative RT-PCR (qRT-PCR)

**Fig. 10:**
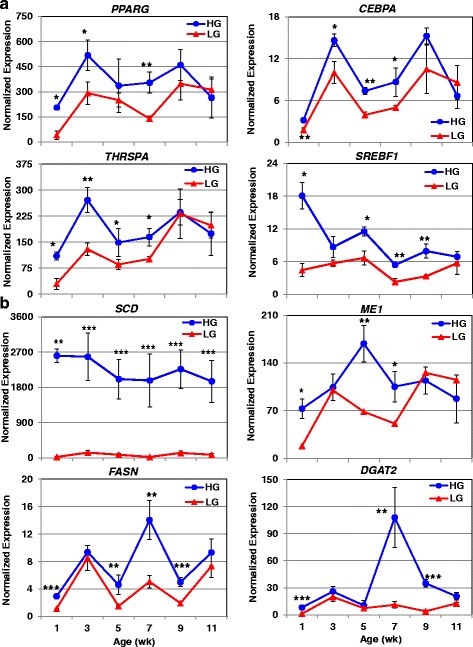
Verification of differentially expressed genes associated with abdominal fatness by qRT-PCR analysis. The abundance of eight genes, expressed higher in the HG and associated with their increased fatness [4 transcription factors (**a**) and 4 metabolic enzymes (**b**)], was verified by qRT-PCR analysis. Data points represent the mean ± SE of 4 birds/genotype and age. Significant differences between genotypes at each age were determined using a one-way ANOVA and Tukey’s multiple comparisons procedure at a significance level of *P*≤0.05 (*), *P*≤0.01 (**) and *P*≤0.001 (***)

Based on biological function, candidate DE genes were selected from the microarray (1-11 wk) and RNA-Seq (7 wk) analyses for verification of expression by qRT-PCR assay. Three genes (*ME1, DIO3*, and *scGH*), not identified by either microarray or RNA-Seq analysis, were also examined by qRT-PCR analysis due to special interest. Expression patterns of four transcriptional regulators which directly regulate adipogenesis and/or lipogenesis are shown in Fig. 10-a. Three of these transcriptional regulators [*PPARG*, *CEBPA* and thyroid hormone responsive spot 14, alpha (*THRSPA*)] show very similar patterns of expression, being up-regulated in HG chickens. Similarly, *SREBF1* was 4-fold higher in HG chickens at 1 wk. (*P* ≤ 0.01), although not different at 3 and 11 wk. Four targets of these transcriptional regulators, controlling lipogenesis, are shown in Fig. 10-b. Interestingly, *SCD* was the second highest-expressed gene identified in abdominal fat of HG chickens by time-course (1-11 wk) microarray analysis (Table 2), being over ~110- and ~90-fold higher (*P*≤0.001) at 1 and 7 wk., respectively. Malic enzyme 1, NADP(+)-dependent, cytosolic (*ME1*) was up-regulated in the HG at 1, 5 and 7 wk., with a peak difference at 5 wk. (~2.5-fold increase in HG chickens). Both *FASN* and *DGAT2* were also significantly up-regulated in HG chickens (*P*≤0.001) at 1, 7 and 9 wk., with the greatest fold-change observed at 7 wk. (2.8- and 9.7-fold, respectively).

**Fig. 11:**
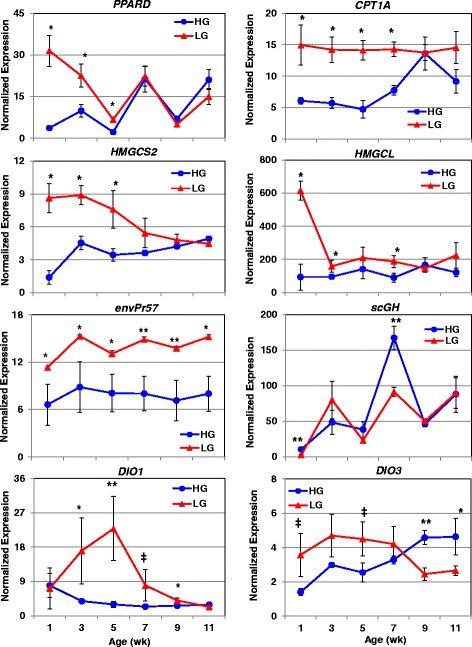
Verification of differential expression of genes associated with leanness by qRT-PCR analysis. The abundance of seven genes, expressed higher in LG birds and associated with leanness, was also verified by qRT-PCR analysis. An additional gene, short isoform of chicken GH (*scGH*), was included in this figure, although its expression was higher in the HG at 7 wk. Data points represent the mean ± SE of 4 birds/genotype and age. Significant differences between genotypes at each age were determined using one-way ANOVA and Tukey’s multiple comparisons procedure at significance levels of *P*≤0.05 (*) and *P*≤0.01 (**). The ‡ symbol denotes a data point that approaches significance (*P*≤0.10)

Transcript abundance was also examined by qRT-PCR for seven genes which appear to be associated with leanness (Fig. 11). The nuclear-hormone receptor *PPARD* (see Fig. 2) was up-regulated in abdominal fat of LG chickens between 1 and 5 wk., with an 8.6-fold difference at 1 wk. Carnitine palmitoyltransferase I (*CPT1A*), which mediates the transport of long chain fatty acids across the outer mitochondrial membrane, was also higher in the LG during early development (1-7 wk) with an average of a 2.4-fold increase across these ages. Two key enzymes in ketogenesis [HMG-CoA synthase 1, soluble (*HMGCS1*) and 3-hydroxy-3-methylglutaryl-CoA lyase (*HMGCL*)] also exhibited early up-regulation in LG chickens. Interestingly, the endogenous avian leukosis virus envelope protein (*envPr57*) was highly expressed in abdominal fat of LG chickens across juvenile development (1-11 wk). The truncated or short chicken growth hormone (*scGH*) transcript was expressed higher (*P*≤0.01) in abdominal fat of HG chickens at 1 and 7 wk. Differences in the thyroid hormone-activating enzyme, deiodinase, iodothyronine, type I (*DIO1*), were seen at 3, 5 and 9 wk. (upregulated in LG chickens at all 3 ages) with the largest difference seen at 5 wk. (7.7-fold higher in LG chickens). Conversely, the gene for the thyroid hormone-deactivating enzyme [deiodinase, iodothyronine, type III (*DIO3*)] was expressed higher in LG chickens at 1 and 3 wk., whereas *DIO3* abundance was greater in visceral fat of the HG at 9 and 11 wk.

The differential expression of 46 genes identified by RNA-Seq analyses at 7 wk. was also examined by qRT-PCR at 7 wk. (Table 4). A subset of 25 DE genes, identified by microarray analysis, was included in the three-way comparison. Eleven of these genes were significantly different (*P* ≤ 0.05) between HG and LG chickens across all three methods (*ALB*, *ALDOB*, *A2M*, *EX-FABP*, *FADS2*, *FGA*, *HSD17B7*, *PDK4*, *SCD*, *THBS2*, and *TTR*). Twelve genes were significantly different (*P* ≤ 0.05) between HG and LG chickens by qRT-PCR, although they did not reach significance level by either RNA-Seq or microarray analyses (*AGTR1*, *ANXA1*, *CEBPA*, *F5*, *FASN*, *HPGDS*, *LDHA*, *LPIN1*, *LPL*, *ND6*, *PPARG*, and *PYGL*). Two genes were not significantly different by qRT-PCR or RNA-Seq analyses, but reached significance in the microarray analysis (*PLG* and *PGRMC1*). Expression ratios of these 25 genes for qRT-PCR analysis versus microarray analysis and RNA-Seq analysis versus microarray analysis comparisons had significant Spearman’s rank coefficients [rho (P) = 0.824615 and 0.854395, respectively]. The additional twenty-two genes analyzed by qRT-PCR at 7 wk. were similar by RNA-Seq analysis in magnitude, direction of fold change and significance level, except for four genes (*GPD1*, *LCN15*, *PDE1C* and *SELP*), which were not significant (*P*≤0.05) by RNA-Seq analysis but reached significance by qRT-PCR analysis. Expression fold-change ratios of forty-seven genes produced a significant Spearman’s rank coefficient [rho (P) = 0.928789] across qRT-PCR and RNA-Seq analyses.

**Table 4:** Differential gene expression in abdominal fat of HG and LG chickens across three analytical methods

	qRT-PCR Analysis		RNA-Seq Analysis		Microarray Analysis	
Gene Symbol	Fold-change	*P*-Value	Fold-change	*P*-Value	Fold-change	*P*-Value
*ALB*	−345.12	1.38E-10	−169.4	5.00E-05	−1.48	0.0100
*ALDOB*	2.75	0.0037	−98.8	5.00E-05	1.4	0.0004
*A2M*	−2.27	0.0412	−52.7	5.00E-05	−5.81	0.0009
*AGTR1*	−2.08	0.0035	−1.93	6.63E-04	−1.92	0.0579
*ANXA1*	3.4	0.0002	2.29	5.00E-05	2.52	0.1303
*CEBPA*	1.73	0.0302	1.44	0.0008	3.19	0.0055
*F5*	−3.21	0.016	−3.24	6.63E-04	−4.34	0.1336
*EX-FABP*	−41.12	1.22E-08	−33.07	6.63E-04	−5.78	0.0069
*FADS2*	4.9	0.0003	4.08	5.00E-05	2.15	0.0004
*FASN*	2.78	0.0033	2.02	5.00E-05	1.35	0.0003
*FGA*	−1929.39	8.46E-11	−282.08	5.00E-05	−7.91	0.0002
*HPGDS*	−2.76	0.0003	−4.57	5.00E-05	−4.05	0.0033
*HSD17B7*	3.38	2.21E-05	3.48	5.00E-05	5.15	0.0401
*LDHA*	−2.74	0.015	−2.02	5.00E-05	−1.58	0.0484
*LPIN1*	2.22	0.0278	1.47	0.0001	1.1	0.5185
*LPL*	3.69	0.0002	2.15	0.0032	1.54	0.0007
*ND6*	−1.78	0.037	−2.55	5.00E-05	−1.28	0.0309
*PPARG*	2.55	0.0047	1.70	5.00E-05	1.66	0.2626
*PYGL*	3.07	0.0068	2.14	0.0833	1.45	0.0052
*PLG*	−7.22	0.0652	−299.71	0.0001	−3.52	0.0493
*PGRMC1*	1.36	0.1159	1.7	1	2.42	0.0205
*PDK4*	−4.95	0.0056	−6.14	5.00E-05	−1.25	0.0002
*SCD*	89.12	0.002	129.4	5.00E-05	3.53	4.52E-13
*THBS2*	3.57	0.0012	2.97	5.00E-05	5.18	2.33E-05
*TTR*	−20.01	9.86E-07	−35.72	5.00E-05	−1.73	0.0229
*DHCR24*	6.18	0.0001	5.46	5.00E-05	-	-
*ACACA*	1.85	0.056	1.82	5.00E-05	-	-
*ACE*	1.91	0.0269	2.47	6.63E-04	-	-
*AGT*	−24.69	1.73E-06	−39.29	6.63E-04	-	-
*APOH*	−24.98	1.48E-06	−122,00	5.00E-05	-	-
*CDS2*	1.99	0.0729	1.81	6.63E-04	-	-
*F8*	−1.49	0.0589	−2.26	6.63E-04	-	-
*DGKQ*	4	1.08E-05	3.18	5.00E-05	-	-
*DPP7*	2.73	0.0003	2.69	0.0086	-	-
*FGB*	−32.54	1.11E-06	−324.53	5.00E-05	-	-
*FGG*	−118.03	0.0016	−197.44	5.00E-05	-	-
*FZD9*	1.54	0.0201	2.07	6.63E-04	-	-
*GPD1*	3.25	6.74E-06	1.92	5.00E-05	-	-
*GREM1*	2.02	0.0007	3.41	5.00E-05	-	-
*IGFALS*	2.13	0.0287	2.49	5.00E-05	-	-
*KLF5*	−3.09	0.0076	−2.70	5.00E-05	-	-
*OSBP2*	2.1	0.0561	2.07	5.00E-05	-	-
*PDE1C*	−1.84	0.0078	−2.04	5.00E-05	-	-
*SELE*	−3.28	0.0139	−7.81	5.00E-05	-	-
*SELP*	−2.08	0.0089	−2.74	5.00E-05	-	-
*SERPINB2*	−6.11	0.0035	−8.87	5.00E-05	-	-

Fold-change values [(+) is higher expression in HG and (−) is higher expression in LG chickens] provided across three independent transcriptional analysis methods at 7 wk. *P*-value for qRT-PCR and microarray analyses were determined by a Student’s T-test on expression values at 7 wk. *P*-value for RNA-Seq is corrected for FDR (FDR ≤ 0.05)

### Independent analysis of two RNA-Seq datasets of abdominal fat at 7 wk. in chickens divergently selected on abdominal fatness (FL vs. LL) or growth rate (HG vs. LG)

**Fig. 12:**
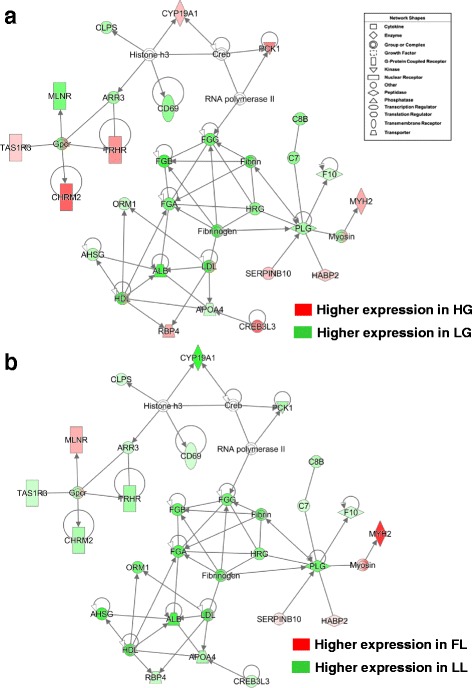
Meta-analysis of RNA-Seq analysis of abdominal fat in 7 wk.-old cockerels, which were genetically selected for a large divergence in either growth rate (HG vs. LG) or abdominal fatness (FL vs. LL), revealed a common network of highly expressed fibrinogenic genes in the leaner lines (LG and LL). This common gene interaction network was identified by IPA from a commonly-shared set of 97 DE genes identified by an independent analysis of visceral fat transcriptomes in HG vs. LG (Panel **a**) and FL vs. LL cockerels (Panel **b**) at 7 wk. The two datasets from RNA-Seq analysis of abdominal fat in the high-growth (HG) versus low-growth (LG) chickens (NCBI GEO Series Accession # GSE49121) and the fat line (FL) versus lean line (LL) broiler chickens (# GSE42980) were downloaded and independently analyzed by the USDA-funded Animal Systems Analysis and Modeling Center (ASBAMC) [48] under an approved project to LAC

The two datasets from RNA-Seq analysis of abdominal fat in the high-growth (HG) versus low-growth (LG) chickens (NCBI GEO Series Accession # GSE49121) and the fat line (FL) versus lean line (LL) broiler chickens (# GSE42980) were independently analyzed by the USDA-funded Animal Systems Analysis and Modeling Center (ASBAMC) [48]. This meta-analysis of abdominal fat transcriptomes across four distinct genotypes revealed 1500 DE genes [adjusted *P*-value (≤0.05) and FDR (≤0.05)] in the HG-LG cockerels and 653 DE genes in the FL-LL at 7 wk. These two datasets were used as input files for “Comparison Analysis” in IPA, which identified 97 commonly shared DE adipose genes. The top over-represented canonical pathways found by IPA were “Acute Phase Response Signaling, Coagulation System, and FXR-RXR Activation”. The top gene interaction network represented by the 97 commonly shared DE genes in HG vs. LG (Fig. 12-a) and FL vs. LL cockerels (Fig. 12-b) was functionally annotated by IPA as “Cardiovascular/Hematological Disease and Developmental Disorder”. This network was composed of a core of fibrinogenic (*FGA, FGB, FGG, HRG,* and *F10),* fibrinolytic (*PLG*) and transporter (*ALB, APOA4, and AHSG*) genes which were highly expressed in leaner chickens from either divergent line (i.e., the LG and LL). However, the expression pattern of several other genes in this network differed according to genetic background. These divergent genes include G-protein coupled receptors (*CHRM2, TRHR, TAS1R3*), estrogen biosynthesis (*CYP19A1*), gluconeogenesis (*PCK1*), retinol transport (*RBP4*), and an upstream regulator of triglyceride metabolism (*CREB3L3*).

## Discussion

Our avian models of growth and metabolism were originally derived from a population of *Bresse-Pile* broiler-type chickens, which were divergently selected by Ricard [23] for either high (HG) or low (LG) body weight (BW) at 8 and 36 wk. Divergent genetic selection on juvenile and adult BW for more than 30 generations has resulted in a 2.7-fold difference in BW of HG and LG cockerels between 1 and 11 wk. (see Fig. 1-a). Perhaps more remarkable was achievement of an even greater difference in visceral fatness, where HG cockerels are 8-fold fatter than the LG birds during juvenile development (Fig. 1-b and c). Thus, the divergent HG and LG chickens serve as unique avian models to unravel the genetic basis of extremes in fatness and leanness, which are incidental to their primary divergence in BW [30, 32]. Divergent genetic selection on abdominal fat alone in the FL and LL chickens has resulted in 2.5-fold difference in visceral fatness at the same body weight [51]. From our current analyses, we found DE genes that were unique to abdominal fat transcriptomes of the HG and LG cockerels, while a common set of DE genes, related to the divergence in abdominal fatness of the HG and LG chickens, was shared with our previous models of divergently selected FL and LL chickens.

For example, our recent transcriptional analyses of abdominal fat in FL and LL chickens revealed over-expression of numerous hemostasis genes in abdominal fat of the leaner LL chickens [21, 22]. The present study has extended this novel finding to slower-growing and leaner LG chickens, which also show over-expression of coagulation and fibrinogenic genes in their diminished visceral fat. Hemostatic processes were amongst the top canonical pathways represented by DE genes identified by RNA-Seq and IPA analyses of abdominal fat in the LG cockerels. We found marked up-regulation of several hemostatic genes in visceral fat of LG chickens that belong to both the intrinsic and extrinsic coagulation pathways (see Fig. 6). Other overexpressed components of the coagulation system and fibrinolysis include serine protease inhibitors that control coagulation (*SERPINA1*, *SERPINB2* and *SERPINF1*) and major transport proteins found in the bloodstream (*A2M*, *ALB*, *GC*, and *TTR*). These plasma transporters could be important in maintaining leanness. For example, A2M is known to inhibit proteases (including coagulation factors) and transport cytokines [52]. Likewise, ALB, GC, and TTR are part of the same family of proteins that transport metabolites associated with leanness including: steroids, fatty acids, vitamin D [53], thyroid hormone [54] and retinoid [55]. Numerous studies of obese humans and murine models have demonstrated direct links between hemostasis and development of metabolic disorders (reviewed in [56–62]). Obese mammals present with a prothrombotic and hypercoagulable state, which is marked by elevated levels of thrombin in circulation. Enhanced production of inflammatory cytokines and adipokines in abdominal fat of obese mammals leads to insulin resistance and impaired fibrinolysis [60, 62]. In contrast, we have discovered over-expression of numerous coagulation genes and “ectopic” endocrine factors in reduced abdominal fat of LL chickens, but not the FL [21, 22]. We have proposed that these highly expressed coagulation factors (mainly proteases and protease inhibitors) could serve a novel role within visceral fat of genetically lean chickens by controlling the proteolytic activation or deactivation of adipokines or other endocrine factors. The present transcriptional analysis of abdominal fat in divergently selected HG and LG chickens clearly shows up-regulation of several hemostatic genes in the diminished visceral fat of the leaner and slower-growing LG chickens, which validates our idea that the enhanced expression of prothrombic and fibrogenic genes in genetically leaner (LL or LG) chickens contributes to their extreme leanness. Furthermore, we have no indication that enhanced expression of coagulation factors in visceral fat of genetically lean chickens would adversely affect systemic hemostasis, which depends upon hepatic synthesis of most clotting precursors. A recent study of three breeds of chickens with distinct differences in abdominal fatness indicates that increased lipid catabolism and reduced lipid synthesis within adipose tissue largely determine leanness in the chicken [63].

The independent meta-analysis of two RNA-Seq datasets from abdominal fat of the HG and LG lines versus the FL and LL lines at 7 wk. has revealed a common gene interaction network of 97 shared genes that are involved in blood coagulation, endocrine signaling, and energy metabolism (Fig. 12). The leaner (LG and LL) chickens over-express a core set of prothrombotic (*FGA, FGB, FGG, F10* and *HRG*) genes, the fibrinolytic gene (*PLG*), acute-phase proteins (*AHSG* and *ORM1*), and several transporter (*ALB, APOA4, HDL* and *LDL*) genes in their diminished abdominal fat. This common “core” network also contains a clusters of divergently-expressed genes related to G-protein-coupled signaling (*CHRM2, TRHR, MLNR* and *TAS1R3*). The sweet taste receptor (*TAS1R3*) was expressed higher in abdominal fat of HG cockerels; whereas, the *TAS1R3* was up-regulated in abdominal fat of the LL chickens at 7 wk. Previously, we found by microarray analysis that the sweet taste receptor (*TAS1R1*) was expressed higher in the hypothalamus of FL chickens at 1 wk. [64], although in visceral fat the *TAS1R1* was over-expressed in the LL chickens [21]. Furthermore, *TAS1R3* knockout mice fed an adipogenic diet present with decreased adiposity and increased bone mass [65]. However, the mechanism by which the sweet taste receptors contribute to diet induced obesity in mice was not elucidated. Clearly, the role of sweet taste receptors (*TAS1R1* and *TAS1R3*) in regulation of energy utilization and adiposity of the chicken and other avian species will require further investigation.

The Del-Mar 14 K Chicken Integrated Systems microarray was used for time-course gene expression profiling in abdominal fat of HG and LG cockerels (1-11 wk). In particular, this custom chicken microarray [30, 31] was well suited for functional gene discovery in abdominal fat, since one-quarter (26%) of cDNAs printed on this 18 K-feature array were derived from abdominal fat cDNA clones. Similar to our transcriptional analyses of fat accretion in the divergent FL and LL cockerels [22], RNA-Seq analysis of abdominal fat was also completed on four HG and four LG cockerels at 7 wk., which was the age with the largest divergence in body weight and visceral fatness. Our time-course microarray analysis clearly shows engagement of several pairs of DE transcription factors in genetic divergence of growth and abdominal fatness traits in HG and LG cockerels. For example, PPARG has a larger number of direct gene targets that support increased synthesis and storage of fatty acids in visceral fat of HG cockerels, while PPARD exerts an opposing action by inhibiting lipid synthesis (*SCD* and *FASN*), while promoting lipolysis and fat catabolism in the LG chickens. RNA-Seq analysis revealed two additional opposing pairs of up-stream regulators [*SREBF1* vs. *VDR* and *SREBF2* vs. *AR*], where the AR and VDR seems to promote expression of catabolic genes and activation of the acute phase response in the LG cockerels. In contrast, other pairs of transcription factors [*SREBF1* vs. *SREBF2;* and *CEBPA* vs. *CEBPB*] seem to be complementary or even synergistic in promoting lipogenesis and angiogenesis in abdominal fat of HG cockerels. Interestingly, the *PGR* appears to act similar to *ESR1* in promoting growth and fat accretion in the HG cockerels, whereas PPARD, AR and VDR seem to support slower growth and diminished visceral fat in the LG cockerels.

The differential expression of numerous transcription factors and their direct target genes in abdominal fat of juvenile HG and LG cockerels could favor either the fast-growing fatter phenotype (HG) or the slower-growing leaner LG phenotype (see Additional files 6 and 7). The major DE upstream regulators that contribute to increased bodyweight and abdominal fatness phenotype of HG chickens include *PPARG, SREBF1, SREBF2*, *CEBPA, E2F4, FOXO1, MSC*, *KLF9, KLF13* and *THRSPA.* On the other hand, the up-regulated transcription regulators that support reduced bodyweight and diminished visceral fatness of the LG phenotype were *PPARD, CEBPB, CALR, PITX2, CREM, STAT5B, AR, PGR, FBXW7, KLF5, MYC* and *NKX2-1*.

Divergent selection for slow growth in meat-type chickens (i.e., LG) substantially inhibits fat deposition, while enhancing catabolism of abdominal fat (see Fig. 1). The most remarkable changes found in expression of abdominal fat genes appear early in post-hatching development of the LG chickens (1-5 wk.; see Fig. 11). The long-chain fatty acid transporter (*SLC27A1*) was upregulated in abdominal fat of LG chickens throughout juvenile development. This gene is highly expressed in human adipose tissue and muscle and much lower in tissues with lower metabolic activity, and is barely detectable in liver [66]. The SLC27 proteins are responsible for the cellular up-take of long chain fatty acids for storage [67] or, more likely, for β-oxidation and enhanced energy production in LG chickens. Correspondingly, the rate-limiting step of β-oxidation is controlled by *CPT1A*, which is highly expressed in abdominal fat of LG chickens. The over-expression of *CPT1A* is driven by increased energy expenditure in chickens, where hepatic expression is up-regulated by fasting [68] or down-regulated after the embryo-hatch transition [32]. Furthermore, several single nucleotide polymorphisms were identified in *CPT1A* of Yup’ik Eskimos, which exhibit fasting-lipid and obesity phenotypes [69]. After transport of long chain fatty acids to the mitochondria, several steps of β-oxidation are catalyzed by the tri-functional protein hydroxyacyl-CoA dehydrogenase (HADH), which produces acetyl-CoA for entry into the TCA cycle. Both subunits of the tri-functional protein (*HADHA* and *HADHB*) were over expressed in LG chickens from 1 to 5 wk., which corresponds to increased expression of mitochondrial malic enzymes (*ME2* and *ME3*) and several subunits of enzyme complexes in the electron transport chain. Although most long-chain fatty acids undergo mitochondrial β-oxidation, several substrates including very-long-chain fatty acids, polyunsaturated fatty acids and several others must be broken down by peroxisomal β-oxidation. The transcript for a major enzyme involved in peroxisomal β-oxidation, *HSD17B4*, was also upregulated in abdominal fat of LG chickens. Deficiency of this enzyme in humans causes a severe developmental syndrome due to cellular build-up of long chain fatty acids and leads to death soon after birth; whereas, knockout of the *HSD17B4* gene in mice blocks the peroxisomal β-oxidation of long chain fatty acids, without causing neonatal death [70].

Ultimately, the TCA cycle appears to be unable to handle the overload of acetyl-CoA produced by β-oxidation of fatty acids in abdominal fat of LG chickens, causing the over-expression of *HMGCS2* and *HMGCL* (see Fig. 11), the two main enzymes in mitochondrial ketone body synthesis. The expression of *HMGCS2* transcripts is greatly induced by fasting in the liver of suckling piglets [71] and 4-week old chickens [68]. In humans, both *HMGCS2* and *HMGCL* exhibit alternative splicing which could regulate their tissue-specific expression [72]. Whether or not this complex regulation exists among different tissues in chickens has not been determined. Proprotein convertase subtilisin/kexin type 1 (*PCSK1*) is another candidate gene, whose expression was 2.2-fold higher in visceral fat of the LG cockerels (Additional file 5). Inhibitors of another members of this gene family, PCSK9, show great promise as a new therapeutic intervention to control hypercholesterolemia and promote cardiovascular health in humans [73]. Taken together, these findings support the idea that LG chickens increase cellular fatty acid up-take, peroxisomal β-oxidation, and transport to/into the mitochondria for β-oxidation and usage in the TCA cycle during early (1-5 wk) development, driving their divergence in abdominal fatness.

The LG chickens appear to down-regulate the mitochondrial breakdown of pyruvate through up-regulation of *PDK4,* which inhibits the pyruvate dehydrogenase (PDH) complex. PDH activity increases with increasing skeletal muscle stimulation (increasing contraction intensity) to a much greater extent in *PDK4* knockout mice than in controls, which suggests that PDK4 is essential for preventing over-activation of the PDH complex [74]. Along with exercise, *PDK4* is over-expressed in rodents subjected to high fat diet or fasting [75], which is similar to what is seen in chickens where both fasting and acute insulin immunoneutralization cause up-regulation when compared to the fed controls [76]. Furthermore, PDK4 appears to be crucial pre-hatch for the acquisition of stored energy, since its expression is quite high between embryonic day e16 and e20, then drops dramatically at hatch and remains low from day 1 through 9 post hatch [32].

Several transcriptional modulators (*RELA, CALR, FBXW7, NR1I3, NR4A3* and *PIAS1*) inhibit transcription of genes involved with increased adiposity (*SCD*, *FASN*, *DGAT2*, *FABP4*, *PCK1*, *LPL*, *PPARG*, *CEBPA*, etc.; discussed below), while activating genes associated with leanness (*COX1*, *HSD17B4*, *DIO1*, etc.). For example, PIAS1 suppresses LXR activation of fatty acid synthesis in murine hepatocytes [77] and NR1I3 is associated with hepatic ω-fatty acid oxidation in quercetin-fed mice [78]. Gene expression of type 1 iodothyronine deiodinase **(** *DIO1*), the major enzyme responsible for the conversion of the pro-hormone T_4_ to metabolically-active T_3_, and *DIO3*, which degrades active T_3_ and converts T_4_ to inactive reverse T_3_ (rT_3_) [79] were examined by qRT-PCR analysis (see Fig. 11). Interestingly, *DIO1* appears to be over-expressed in LG chickens during early juvenile development (3-7 wk), while expression of the degrading enzyme *DIO3* is higher in HG chickens at later ages (9 and 11 wk). This could provide higher levels of T_3_ in abdominal fat of LG chickens throughout juvenile development, which would contribute to their leanness [54]. The nuclear hormone receptor PPARD (see Fig. 2) has similar activation and inhibition targets and directly up-regulates several additional genes associated with leanness (i.e., *ANGPTL3*, *PLP1* and *PDK4*). Selective knock-in of *PPARD* in adipose tissue of Lep^db/db^ mice reduces lipid accumulation and prevents obesity in mice [80]. Early (1 to 5 wk) up-regulation of *PPARD* in abdominal fat of LG chickens and its direct action on target genes suggest that PPARD is a major regulator of lipolysis in these leaner and slower growing animals.

Presently, we show that HG cockerels over-express many genes controlling adipogenesis and lipogenesis in visceral fat (see Figs. 2 and 4; Table 4), which seems against the convention that abdominal fat only makes a minimal contribution to lipogenesis in the chicken [81]. This finding is supported by our earlier studies that show enhanced expression of genes controlling these processes in abdominal fat of fat line (FL) chickens [21]. Upregulation of adipogenesis and lipogenesis in abdominal fat of HG chickens is likely supported by increased activity and interaction of several transcription factors, including PPARG [82], CEPBA [83], SREBF1 [84] and THRSPA [85, 86]. These transcriptional regulators are over-expressed throughout juvenile development and control common lipogenic targets, including *DGAT2*, *FADS2*, *FASN* and *SCD* (discussed below). Furthermore, IPA predicts the up-regulation of an additional 13 transcriptional regulators associated with fatness of HG chickens, which also directly increase the expression of lipogenic genes and other genes associated with fatness (see Additional files 6 and 7). Microarray analysis of liver in transgenic mice over-expressing *SREBF1* or *SREBF2* show that they have many common targets and both regulators play a crucial role in synthesis and metabolism of fatty acids and cholesterol [87]. Currently, our microarray and RNA-Seq analyses of abdominal fat in the HG and LG chickens show that SREBF1 and SREBF2 support increased growth rate and abdominal fatness of HG cockerels. These ligand-activated transcription factors enhance lipogenesis as indicated by their over-expression in HG chickens and direct redundant activation of numerous target genes controlling lipid metabolism (*CYB5A, DHCR7, FADS2, FASN, INSIG1, LSS, MSMO1, PCYT1A, SC5D, SCD* and *SQLE*) (see Fig. 7-b).

*INSIG1*, also higher in HG chickens, may be a major regulator of fatty acid and cholesterol synthesis through the direct regulation of the SREBF chaperone (SCAP), and HMG-CoA reductase [88]. Two additional genes involved in cholesterol synthesis (*DHCR24* and *HSD17B7*) are commonly up-regulated by divergent selection for high body weight or high abdominal fatness [21]. The expression of *DHCR24* in whole blood was highly correlated and decreased with weight loss after bariatric surgery in humans [89]. Two other genes which are over-expressed in adipose tissue of HG chickens, *ACACA* and *ACSS2*, were identified as predictors of weight loss in another group of patients who underwent bariatric surgery, where decreased expression in adipose tissue was associated with a decrease in weight and hip circumference [90]. Acetyl CoA carboxylase (*ACACA* or *ACC1*) is critically important for the generation of malonyl CoA and synthesis of long chain fatty acids. This was demonstrated in human hepatoma HepG2 cells where inhibition of ACC1 by soraphen A reduces de novo lipogenesis by attenuating the formation of malonyl CoA and long chain fatty acids [91]. Overexpression of fatty acid elongases, *ELOVL5* and *ELOV6*, or desaturases, *FADS1* and *FADS2* were not successful in reversing the effect of soraphen A on production of long chain fatty acids, which supports a crucial role of ACC1.

Perhaps one of the most significant findings of abdominal fat transcriptomes in the HG and LG chickens was a very large difference in expression of *SCD*, the rate-limiting enzyme responsible for conversion of saturated fatty acids into monounsaturated fatty acids. At 7 wk., *SCD* was the highest-expressed gene found in abdominal fat of HG chickens (see Fig. 10-b), where its expression was approximately 140-fold greater than that of the LG. This large difference was present throughout juvenile development (1-11 wk), where *SCD* transcripts were nearly undetectable in abdominal fat of LG chickens. Mutation of the *SCD* gene in mice produces a lean and hyper-metabolic phenotype, while *ob/ob* mice that also carrying mutations in *SCD* exhibit reduced fatness and elevated metabolism [92]. Another desaturase expressed very highly in abdominal fat of HG chicken is *FADS2*, which is down-regulated in liver in response to fasting in both pigs [93] and chickens [68], as well as in the pre-hatch chick embryo [32]. The major multi-enzyme protein responsible for the synthesis of fatty acids, *FASN*, is also very highly expressed in HG chickens at several ages, with the peak difference observed at 7 wk., the age of maximal distinction in abdominal fat between the two genotypes. The essential role of *FASN* in the fatty acid biosynthesis is highlighted in mouse embryos where *FASN* knockout mice die before birth and heterozygous knockout mice die at various stages of development [94]. An incredibly similar expression pattern (as *FASN*) was observed for *DGAT2*, which was almost 10-fold higher in HG chickens at 7 wk. DGAT2 catalyzes the final committed step in triacylglycerol synthesis and is critical for formation of adipose tissue [95, 96]. Overexpression of *DGAT2* in mammalian HEK293 cells significantly increases triglyceride (TG) synthesis while inhibition of *DGAT2* by compound 122 decreases TG synthesis in a dose dependent manner [97]. Further, *DGAT2* is up-regulated in goose hepatocytes exposed to a mixture of long chain fatty acids [98]. Correspondingly, *ACSL1*, which generates fatty acyl-CoA for entry into the triacylglycerol synthesis pathway, and *AGPAT9,* which catalyzes the conversion of glycerol-3-phosphate to lysophosphatidic acid, the initial step of triacylglycerol synthesis [99] were both overexpressed in HG chickens. Abdominal fat of HG chickens also over expresses *GPD1*, which serves as a link between carbohydrate metabolism and lipid metabolism by catalyzing the conversion of dihydroxyacetone phosphate to glycerol-3-phosphate, the major component of glycerophospholipids. In fact, HG chickens up-regulate several genes involved in glycolysis including hexokinase 1 (*HK1*), which phosphorylates glucose to produce glucose-6-phosphate, the first step in the glycolytic pathway, and *ALDOB*, which converts fructose 1,6-bisphosphate into glyceraldehyde 3-phosphate and dihydroxyacetone phosphate (substrate for *GPD1*). Taken together, HG chickens up-regulate genes required for production of acetyl-CoA used in biosynthesis of fatty acids, cholesterol and triglycerides, which are highly upregulated processes in the abdominal fat of these animals.

Another important observation from the present transcriptional study was the predominate overexpression of avian leukosis virus (*ALVE* or *envPr57* polyprotein) transcripts in the LG chickens across all ages (1-11 wk) and tissues (pituitary, abdominal fat, liver and breast muscle) examined [32]. The abundant overexpression of endogenous *envPR57* transcripts in abdominal fat of LG cockerels during juvenile development (1-11 wk) was verified by qRT-PCR analysis (Fig. 11). It is of particular interest that *ALVE* transcripts are also highly-expressed in the hypothalamus [10], brain and peripheral tissues of the low-weight strain (LWS) chickens developed at Virginia Tech, particularly in immature pullets [6, 11], where a greater number of *ALVE* integration sites are found in LWS pullets when compared to high-weight (HWS) females. These authors concluded that *ALVE* sequences might directly affect growth or could be linked to loci regulating growth. The relationship between the multiple *ALVE* loci and genetic selection of production traits was examined in several meat-type chickens [100, 101]. Although our HG and LG lines were divergently selected from the *Bresse-Pile* breed in France [23] and the Virginia HWS and LWS lines were independently selected from the White Plymouth Rock breed. It is not known whether the higher abundance of *ALVE* in the low-growth (and low abdominal fatness) lines could reflect inheritance from a common ancestor or a direct response of selection for low growth. It is possible that the high frequency of *ALVE* insertion sites in the genome of meat-type chickens could disrupt key functional genes controlling growth or metabolism traits.

Lastly, we found evidence for a divergence in growth hormone (GH) signaling in the divergently selected HG and LG cockerels. Plasma levels of pituitary GH are elevated 2.5-fold in LG chickens, whereas plasma IGF-1 levels are 3-fold higher in the faster-growing and fatter HG birds (Duclos MJ, Simon J and Cogburn LA; unpublished observations). The LG cockerels also exhibit diminished GH binding to hepatic GH receptors (GHR), which seems to reflect the subsequent lack of negative feedback control on GH secretion from the pituitary gland. Thus, the reduced growth rate of LG chickens reflects a disruption in GHR signaling as evidenced by a reduction in hepatic synthesis of IGF-I and low plasma IGF-I levels [29]. Presently, we found a greater abundance of the small chicken GH (*scGH*) in abdominal fat of HG chickens at 7 wk. when compared to LG birds (see Fig. 11). The *scGH* was originally identified in the eye of chick embryos [102] and apparently exerts a local action in ocular tissue via an autocrine or paracrine action, since the signal sequence is absent [103–105]. Earlier, we identified a similar truncated *cGH* transcript in adipose tissue of the chicken from an exhaustive expressed-sequence tag (EST) sequencing project [106]. Insulin-like growth factor (IGF) signaling could be up-regulated in visceral fat of HG chickens, since *IGFBP2* is over-expressed in abdominal fat of HG birds. Interestingly, IGFBP2 is primarily secreted from adipocytes and diminishes with childhood obesity [107]. Clearly, further studies on GH and IGF signaling in chicken adipose tissue are needed to fully understand how GH and IGF-1 contribute to lipogenesis and adiposity of chickens. Our earlier studies clearly show that exogenous GH increases abdominal fatness in juvenile chickens without affecting growth rate or plasma IGF-1 levels [108–112], which is unlike the typical mammalian response to exogenous GH [113–115].

## Conclusions

Divergent selection for bodyweight at two ages (juvenile and adult) in meat-type chickens has profound effects on abdominal fatness and body weight traits (phenotypes). Throughout juvenile development, visceral fat of HG chickens over-express several transcription factors, which promote lipogenesis and adipogenesis. These regulators of transcription appear to be responsible for the up-regulation of several processes (i.e., biosynthesis of fatty acids, cholesterol and triglycerides) that increase abdominal fatness in HG cockerels. These lipogenic genes are not only differentially expressed, but are also among the most abundant transcripts found in abdominal fat of the HG. During early juvenile (1-5 wk) development, the slower growing and leaner LG chickens increase expression of several genes that promote energy expenditure, which likely contributes to their extreme leanness (i.e., oxidative phosphorylation, peroxisomal β-oxidation, mitochondrial β-oxidation and ketogenesis). Also, the expression of functional energy-consuming genes could be altered by the ALVE protein itself or random viral *ALVE* insertions, found throughout the chicken genome, could disrupt gene expression. Up-regulation of hemostatic factors (proteases and protease inhibitors) appears critical to development of extremes in leanness found in LG and LL chickens derived from different genetic backgrounds. This lends support to the idea that *genetic* selection for a divergence in growth or fatness traits also provides insight into mechanisms controlling fatness and leanness phenotypes. Meta-analysis of two RNA-Seq datasets from abdominal fat in divergently selected chickens from two distinct genetic backgrounds provides novel evidence that adipose genes encoding coagulation proteins are associated with the lean phenotype in meat-type chickens. While the exact mechanisms by which hemostatic genes contribute to leanness in chickens have not been clearly defined, the present transcriptional study of abdominal fat in HG and LG cockerels validates our previous reports that describe involvement of the hemostasis system in limiting adiposity of the leaner (LL) chickens [21, 22]. Furthermore, the present study demonstrates that abdominal (visceral) fat of the chicken is a dynamic metabolic and endocrine tissue, which has an unappreciated capacity for in situ lipogenesis.

## Additional files

Additional file 1: Microarray experimental design. A Microsoft Excel file containing a single work sheet “Array Hybridization Design” which described the hybridization scheme for the HG and LG adipose tissue microarray experiment. (XLSX 13 kb)

Additional file 2: Primer information. A Microsoft Excel file containing a single work sheet “Primer information”. For each primer used for qRT-PCR analysis, gene symbol, gene name, forward and reverse primer sequences, GenBank accession, and amplicon size (bp) are provided. (XLSX 20 kb)

Additional file 3: Differentially expressed (DE) genes identified by time-course microarray analysis of abdominal fat in HG and LG cockerels (1-11 wk). A Microsoft Excel file containing three worksheets representing the main effect of genotype (*G*), the main effect of age (*A*), and the *G* x *A* interaction. The *G, G* x *A* interaction and *combined unique* (*G* plus *G* x *A* gene lists) datasets were used for Ingenuity Pathway Analysis (IPA). (XLSX 484 kb)

Additional file 4: Power analysis of the HG and LG abdominal fat RNA-Seq dataset. (A) The publically available web-based software program called “Scotty” [49, 50] was used for a power analysis to demonstrate adequate biological samples size and sequencing depth. The power of detection was calculated at ≥1.5, 2, or 3-fold change differences between HG (*N* = 4) and LG (*N* = 4) chickens at a significance level of *P*≤0.05 and >50 M reads per biological sample. We achieved the power to detect 70% genes with a ≥1.5-fold difference as indicated by the red dashed line. (**B**) The “Scotty” program also provided a hierarchical cluster analysis using the Spearman correlation as the distance metric to demonstrate relatedness among the eight individual (4 HG and 4 LG) birds used for RNA-Seq analysis of abdominal fat at 7 wk. (PPTX 352 kb)

Additional file 5: Differentially expressed (DE) (FDR ≤0.05) identified by RNA-Seq analysis of abdominal fat in HG and LG cockerels (7 wk). A Microsoft Excel file containing one worksheet. (XLSX 277 kb)

Additional file 6: Top pathways and biological functions identified by IPA from time-course microarray analysis of abdominal fat in HG and LG cockerels (1-11 wk). A Microsoft Excel file containing eight worksheets [i.e.*,* “*Upstream Regulators, Oxidative Phosphorylation, LXR/RXR Activation, Fatty Acid Metabolism, Adipogenesis Pathway, Insulin Resistance, VEGF Signaling*, and *Protein Metabolism*”]. Top canonical pathways and biological functions were identified by Ingenuity Pathway Analysis (IPA) of 647 “Analysis Ready” (AR)-DE genes (FDR ≤ 0.05) from time-course (1-11 wk) microarray analysis. (XLSX 49 kb)

Additional file 7: Top canonical pathways and biological functions identified by Ingenuity Pathway Analysis of RNA-Seq analysis of abdominal fat in HG and LG cockerels (7 wk). A Microsoft Excel file containing 10 worksheets of the DE genes assigned by Ingenuity Pathway Analysis to top canonical pathways and biological functions [i.e.*,* “*Upstream Regulators, Acute Phase Signaling, Coagulation System, Intrinsic Prothrombin Activation, Extrinsic Prothrombin Activation, LXR/RXR Activation, Fatty Acid metabolism, Adipogenesis Pathway, Insulin Resistance, VEGF Signaling*, and *Protein Metabolism*”]. (XLSX 81 kb)
