# Supplementary material for: Transcriptional analysis of abdominal fat in chickens divergently selected on bodyweight at two ages reveals novel mechanisms controlling adiposity: validating visceral adipose tissue as a dynamic endocrine and metabolic organ
Source: BMC Genomics. 2017 Aug 16;18:626. doi: 10.1186/s12864-017-4035-5 (PMC5559791; doi:10.1186/s12864-017-4035-5)
Supplement: Supplementary file 4 — Power analysis of the HG and LG abdominal fat RNA-Seq dataset. (A) The publically available web-based software program called “Scotty” [49, 50] was used for a power analysis to demonstrate adequate biological samples size and sequencing depth. The power of detection was calculated at ≥1.5, 2, or 3-fold change differences between HG (N = 4) and LG (N = 4) chickens at a significance level of P≤0.05 and >50 M reads per biological sample. We achieved the power to detect 70% genes with a ≥1.5-fold difference as indicated by the red dashed line. (B) The “Scotty” program also provided a hierarchical cluster analysis using the Spearman correlation as the distance metric to demonstrate relatedness among the eight individual (4 HG and 4 LG) birds used for RNA-Seq analysis of abdominal fat at 7 wk. (PPTX 352 kb) [file 12864_2017_4035_MOESM4_ESM.pptx]

## Slide 1
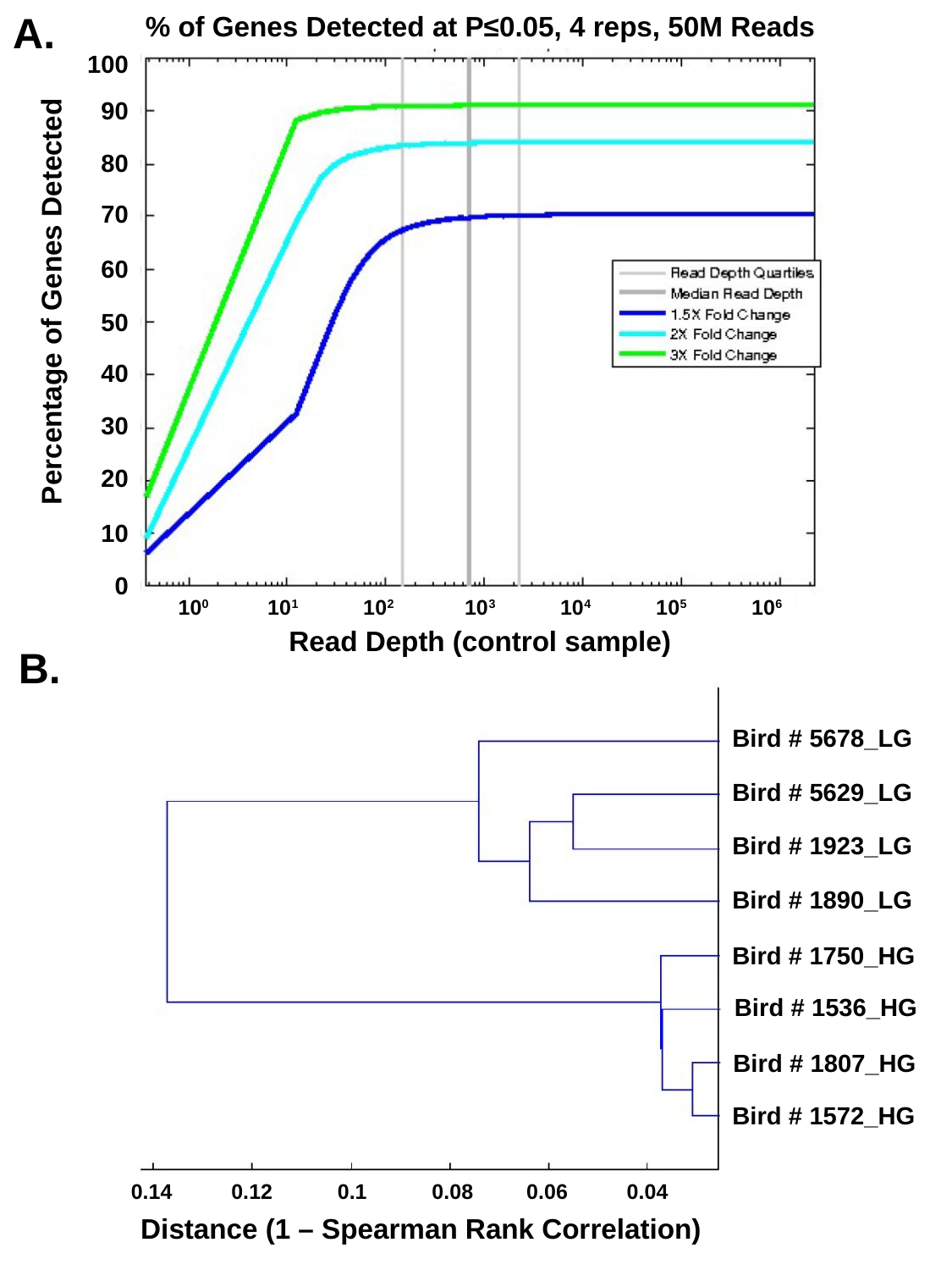

A.
% of Genes Detected at P≤0.05, 4 reps, 50M Reads
100
90
80
70
60
50
40
30
20
10
0
Percentage of Genes Detected
100 101 102 103 104 105 106
Read Depth (control sample)
B.
Bird # 5678_LG
Bird # 5629_LG
Bird # 1923_LG
Bird # 1890_LG
Bird # 1750_HG
Bird # 1536_HG
Bird # 1807_HG
Bird # 1572_HG
0.14 0.12 0.1 0.08 0.06 0.04
Distance (1 – Spearman Rank Correlation)
